# Stretch-activated current in human atrial myocytes and Na^+^ current and mechano-gated channels’ current in myofibroblasts alter myocyte mechanical behavior: a computational study

**DOI:** 10.1186/s12938-019-0723-5

**Published:** 2019-10-25

**Authors:** Heqing Zhan, Jingtao Zhang, Anquan Jiao, Qin Wang

**Affiliations:** 1 College of Medical Information, Hainan Medical University, Haikou, 571199 China; 2 Cardiac Arrhythmia Center, Fuwai Hospital, National Center for Cardiovascular Diseases, Beijing, 100037 China

**Keywords:** Mechano-gated channels (MGCs), Myocyte mechanics, Mathematical modeling, Myofibroblast–myocyte (Mfb–M) coupling, Stretch-activated channels (SACs), Voltage-gated sodium channels (VGSCs)

## Abstract

**Background:**

The activation of stretch-activated channels (SACs) in cardiac myocytes, which changes the phases of action potential repolarization, is proven to be highly efficient for the conversion of atrial fibrillation. The expression of Na^+^ current in myofibroblasts (Mfbs) regenerates myocytes’ action potentials, suggesting that Mfbs play an active role in triggering cardiac rhythm disturbances. Moreover, the excitation of mechano-gated channels (MGCs) in Mfbs depolarizes their membrane potential and contributes to the increased risk of post-infarct arrhythmia. Although these electrophysiological mechanisms have been largely known, the roles of these currents in cardiac mechanics are still debated. In this study, we aimed to investigate the mechanical influence of these currents via mathematical modeling. A novel mathematical model was developed by integrating models of human atrial myocyte (including the stretch-activated current, Ca^2+^–force relation, and mechanical behavior of a single segment) and Mfb (including our formulation of Na^+^ current and mechano-gated channels’ current). The effects of the changes in basic cycle length, number of coupled Mfbs and intercellular coupling conductance on myocyte mechanical properties were compared.

**Results:**

Our results indicated that these three currents significantly regulated myocyte mechanical parameters. In isosarcometric contraction, these currents increased segment force by 13.8–36.6% and dropped element length by 12.1–31.5%. In isotonic contraction, there are 2.7–5.9% growth and 0.9–24% reduction. Effects of these currents on the extremum of myocyte mechanical parameters become more significant with the increase of basic cycle length, number of coupled Mfbs and intercellular coupling conductance.

**Conclusions:**

The results demonstrated that stretch-activated current in myocytes and Na^+^ current and mechano-gated channels’ current in Mfbs significantly influenced myocyte mechanical behavior and should be considered in future cardiac mechanical mathematical modeling.

## Background

As an alternative of experimental studies, computational modeling studies provide a powerful framework for gaining substantial insights of cardiac electrophysiology and mechanics in many aspects [1, 2]. For cardiac electrophysiological simulation, cardiac cell action potential (AP) models are built to represent current flow through ion pumps, channels, and exchangers [3, 4]. For mechanical simulation, the active stress/strain models and Hill’s three-element model have been formulated to lay out active contraction [5, 6].

Cardiac electrical activities have intimate connection with mechanical actions. The interaction between them is referred as electromechanical coupling (EMC) and mechanoelectrical feedback (MEF) [7]. As a major mechanism of the MEF, the stretch-activated channel (SAC) has been used to explain changes in electrophysiological behavior by mechanical deformation. Several cellular experimental and modeling studies have examined the impact of SACs on cardiac electrophysiology [8–11].

Recently, clinical data and simulation studies have provided some important insights into cardiac structural remodeling, especially fibrosis as a hallmark of permanent atrial fibrillation. Many of them verified that both fibroblasts and myofibroblasts (Mfbs) modulated cardiac electrical conduction, coordinated tissue remodeling, and integrated signals [12–14]. They have been considered as active communicators rather than non-excitable cells, which involve several currents like cardiac myocytes, e.g., the currents through potassium channels [15, 16], the non-selective transient receptor potential cationic channel subfamily M member 7 (TRPM7) [17], voltage-gated sodium channels (VGSCs) [18, 19], chloride channels [20], single mechano-gated channels (MGCs) [21], and voltage-dependent proton currents [22].

Computational models of atrial fibrosis have been used to investigate how fibroblasts modulate cardiac myocyte electrophysiology. At the cellular level, processes of fibrotic remodeling are represented as fibroblast proliferation and phenotype switching [23, 24]. Simulation results showed that coupling of fibroblasts or Mfbs to atrial myocytes resulted in shorter duration of the action potential (APD), slower conduction, and spiral wave breakups [25–28].

As a critical determinant of cardiac mechanics, fibroblast-mediated changes in extracellular matrix structure are also investigated by computational modeling. Cell compaction of collagen gels has been studied by explicitly calculating the mechanical equilibrium between each cell’s contractile forces and nearby collagen fibers’ mechanical properties [29, 30]. Infarct mechanics has been simulated by coupling agent-based model predictions to a finite element model [31, 32]. These studies have found that fibroblast alignment parallel to a strain cue provides a negative feedback to radical changes in local fiber orientations.

Previous studies mentioned above have examined cardiac myocyte functions in many aspects; however, no study to the our best knowledge has considered the following two aspects in Mfb–myocyte (Mfb–M) coupling, especially in EMC: (1) the stretch-activated ion channel current (*I*_SAC_) in myocytes, which influences cardiac myocytes electrophysiological characteristics under stretching [33, 34]; (2) the currents through VGSCs (*I*_Na_Mfb_) and MGCs (*I*_MGC_Mfb_) in Mfbs, which could influence Mfb properties and contribute to EMC in cardiac pathologies [18, 21].

Our previous study has found that *I*_Na_myofb_ and *I*_MGC_Mfb_ regenerated APs in myocytes and Mfbs [28]. In this study, we aimed to investigate the role of *I*_SAC_, *I*_Na_Mfb_, and *I*_MGC_Mfb_ in the mechanical contraction of cardiac myocyte. Simulation results of human atrial myocyte segment mechanical dynamics with different gap-junctional conductance (*G*_gap_), number of coupled Mfbs and basic cycle lengths (BCLs) were examined.

## Results

### Effects of *I*_SAC_, *I*_Na_Mfb_, and *I*_MGC_Mfb_ on atrial myocyte AP, [Ca^2+^]_*i*_, and the normalized force

**Fig. 1:**
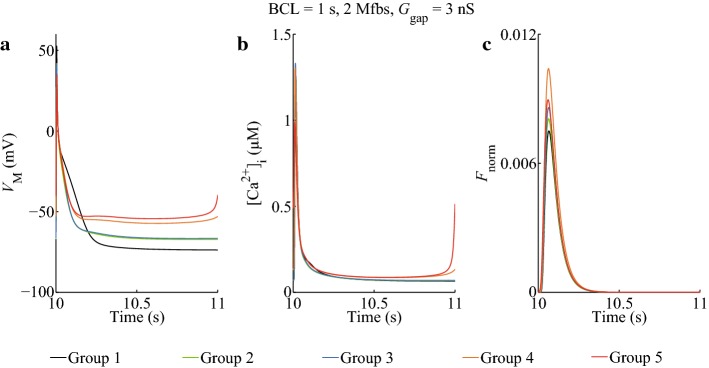
Effects of *I*_SAC_, *I*_Na_Mfb_, and *I*_MGC_Mfb_ on atrial myocyte **a** AP, **b** [Ca^2+^]_*i*_, and **c** *F*_norm_ in five groups. BCL = 1 s, 2 Mfbs, *G*_gap_ = 3 nS

Figure 1 shows the combinational effects in five groups (see “Simulation protocol” section) of *I*_SAC_, *I*_Na_Mfb_, and *I*_MGC_Mfb_ on the membrane potential, intracellular Ca^2+^ concentration and the normalized force (*F*_norm_) of myocytes with a *G*_gap_ of 3 nS and a BCL of 1 s. For myocytes, coupling Mfbs (Group 2-5) resulted in gradual decrease of myocyte membrane potential amplitude (*V*_max_) and APD at 90% repolarization (APD_90_), and increase of the resting myocyte membrane potential (*V*_rest_) depolarization (Fig. 1a). Meanwhile, a spontaneous excitement was emerged in Group 5, in which the peak [Ca^2+^]_*i*_ dropped significantly (Fig. 1b), indicating that *I*_SAC_, *I*_Na_Mfb_, and *I*_MGC_Mfb_ could result in discordant alternans. From the traces of *F*_norm_ (Fig. 1c), it can be observed that the peak *F*_norm_ increased after myocyte coupled to Mfbs. It was increased by 7.6% (Group 2), 14.5% (Group 3), 38.7% (Group 4), and 19.2% (Group 5) as compared to the control (Group 1). It was remarkable that Group 4 got the biggest *F*_norm_ increment, which meant *F*_norm_ of myocytes could be significantly enhanced by the combination of *I*_SAC_ and *I*_Na_Mfb_. However, the increment relatively declined in Group 5 with the introduction of *I*_MGC_Mfb_. This might suggest that deformation in myocytes enhanced *F*_norm_, while in Mfbs the deformation relatively hindered it. The effects of *I*_SAC_ and *I*_MGC_Mfb_ on the force of atrial myocytes were opposite, with *I*_SAC_ increasing and the other one decreasing.

### Effects of *I*_SAC_, *I*_Na_Mfb_, and *I*_MGC_Mfb_ on atrial myocyte segment mechanical parameters

**Fig. 2:**
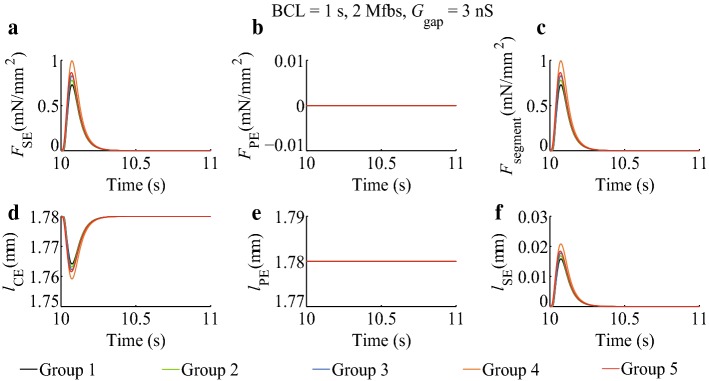
Effects of *I*_SAC_, *I*_Na_Mfb_, and *I*_MGC_Mfb_ on atrial myocyte segment mechanical parameters in isosarcometric contraction in five groups. BCL = 1 s, 2 Mfbs, *G*_gap_ = 3 nS

**Fig. 3:**
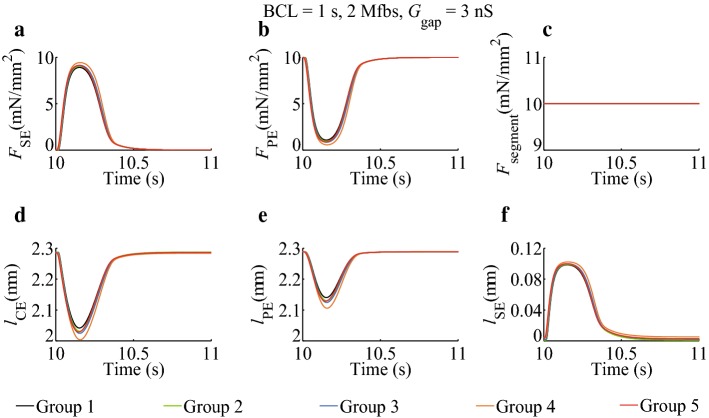
Effects of *I*_SAC_, *I*_Na_Mfb_, and *I*_MGC_Mfb_ on atrial myocyte segment mechanical parameters in isotonic contraction in five groups. BCL = 1 s, 2 Mfbs, *G*_gap_ = 3 nS

Traces of *F*_SE_, *F*_PE_, *F*_segment_, *l*_CE_, *l*_PE_, and *l*_SE_ obtained in five groups for the simulations of isosarcometric contraction with sarcomere length of 1.78 µm are displayed in Fig. 2, and the ones for simulations of isotonic contraction with applied force of 10 mN per square millimeter (mN/mm^2^) are displayed in Fig. 3.

In isosarcometric contraction (Fig. 2), *F*_PE_ and *l*_PE_ in five groups were constant. Peak *F*_SE_ increased when myocyte coupled to Mfbs. It was increased by 7.2% (Group 2), 13.8% (Group 3), 36.6% (Group 4), and 18.5% (Group 5) as compared to the control (Group 1). Using Eq. (14) (see “Mechanical behavior of a single segment” section), the increments in *F*_segment_ were the same as those in *F*_SE_. On the contrary, *l*_CE_ decreased when myocyte coupled to Mfbs. The minimum of *l*_CE_ was dropped by 6.4% (Group 2), 12.1% (Group 3), 31.5% (Group 4), and 16.2% (Group 5) as compared to the control (Group 1). Using Eq. (15), the changes in *l*_CE_ were equal to those in *l*_SE_. Similar to Fig. 1, Group 4 had the most significant change, indicating that the combination of *I*_SAC_ and *I*_Na_Mfb_ played a significant role in determining myocyte segment mechanical behavior.

In isotonic contraction (Fig. 3), *F*_segment_ in five groups was constant. Like Fig. 2a, the peak value of *F*_SE_ also increased when Mfbs were coupled. It was increased by 1.6% (Group 2), 2.7% (Group 3), 5.9% (Group 4), and 2.5% (Group 5) as compared to the control (Group 1). The increments were smaller than those in isosarcometric contraction. According to Eq. (14), the decrements in *F*_PE_ were equal to the increments in *F*_SE_. The minimum of *l*_CE_ was dropped by 4.3% (Group 2), 7.4% (Group 3), 15.8% (Group 4), and 6.3% (Group 5) as compared to the control (Group 1), while the peak value of *l*_SE_ was increased by 0.6%, 1.4%, 3.7%, and 0.9%, and finally, *l*_PE_ were declined by 6.8%, 11.4%, 24.0%, and 10.0%. *I*_SAC_, *I*_Na_Mfb_, and *I*_MGC_Mfb_ not only changed the extreme values of *l*_CE_, *l*_PE_, and *l*_SE_, but also altered *l*_CE_ and *l*_SE_ in the resting stage. For example, the value of *l*_SE_ in resting period was 0.0021 μm (Group 1), 0.0013 μm (Group 2), 0.0021 μm (Group 3), 0.0051 μm (Group 4), and 0.0032 μm (Group 5), respectively. Similarly, Group 4 has the most impact on the mechanical parameters.

**Fig. 4:**
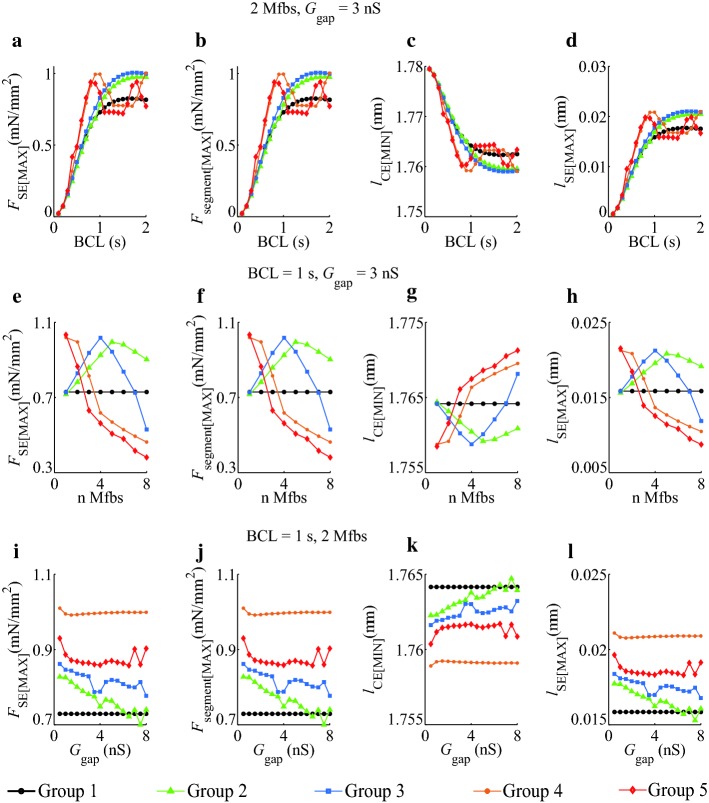
Effects of *I*_SAC_, *I*_Na_Mfb_, and *I*_MGC_Mfb_ on the extremum of *F*_SE_, *F*_segment_, *l*_CE_, and *l*_SE_ as functions of BCL, Mfb–M ratio, and *G*_gap_ in isosarcometric contraction in five groups, **a**–**d** BCL = 0.1–2 s, Mfb:M = 2, *G*_gap_ = 3 nS, **e**–**h** BCL = 1 s, Mfb:M = 1–8, *G*_gap_ = 3 nS, and **i**–**l** BCL = 1 s, Mfb:M = 2, *G*_gap_ = 0.5–8 nS

**Fig. 5:**
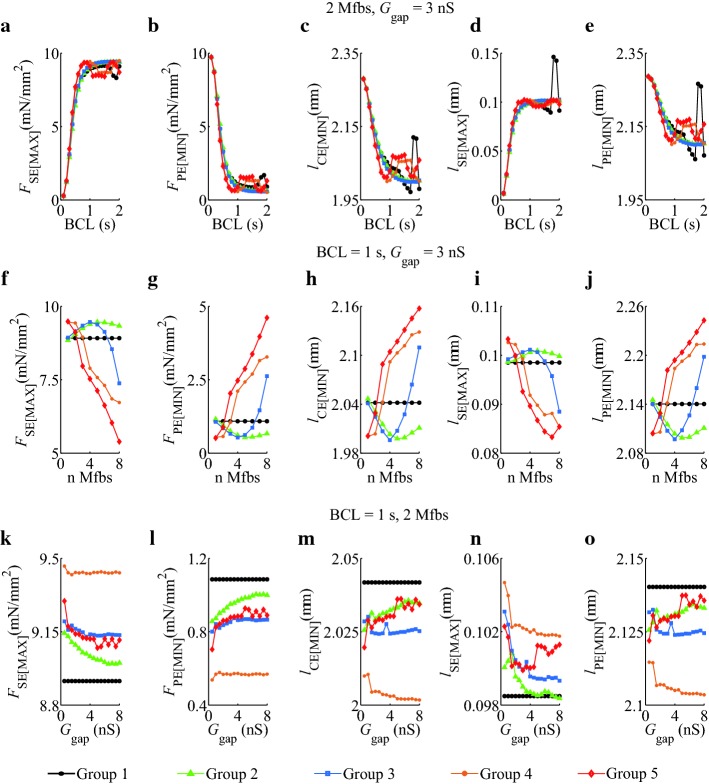
Effects of *I*_SAC_, *I*_Na_Mfb_, and *I*_MGC_Mfb_ on the extremum of *F*_SE_, *F*_PE_, *l*_CE_, *l*_SE_, and *l*_PE_ as functions of BCL, Mfb–M ratio, and *G*_gap_ in isosarcometric contraction in five groups. **a**–**e** BCL = 0.1–2 s, Mfb:M = 2, *G*_gap_ = 3 nS, **f**–**j** BCL = 1 s, Mfb:M = 1–8, *G*_gap_ = 3 nS, and **k**–**o** BCL = 1 s, Mfb:M = 2, *G*_gap_ = 0.5–8 nS

To investigate the effects of *I*_SAC_, *I*_Na_Mfb_, and *I*_MGC_Mfb_ on the extreme values of atrial myocyte segment mechanical parameters, we simulated five groups with different BCLs, Mfb–M ratios, and *G*_gap_ in both isosarcometric contraction (Fig. 4) and isotonic contraction (Fig. 5).

From the traces of the extremum of *F*_SE_, *F*_segment_, *l*_CE_, and *l*_SE_ for BCL = 0.1–2 s (Fig. 4a–d), it could be observed that peak *F*_SE_ (*F*_SE[MAX]_), peak *F*_segment_ (*F*_segment[MAX]_), and peak *l*_SE_ (*l*_SE[MAX]_) increased, and valley value of *l*_CE_(*l*_CE[MIN]_) decreased with increasing BCL, in isosarcometric contraction. When BCL was less than 1 s, *F*_SE[MAX]_, *F*_segment[MAX]_ and *l*_SE[MAX]_ at each BCL increased and *l*_CE[MIN]_ decreased in Groups 2–5 as compared to Group 1. Meanwhile, at each BCL, *F*_SE[MAX]_, *F*_segment[MAX]_, and *l*_SE[MAX]_ in Group 4 reached their maximums, and *l*_SE[MAX]_ obtained its minimum. This suggests that *I*_SAC_ together with *I*_Na_Mfb_ had the key influence on myocyte mechanical parameters. The influence disappeared in Group 5, suggesting that the role of *I*_MGC_Mfb_ in myocytes was opposite compared to *I*_SAC_. As BCL longer than 1 s, each parameter in Group 1 to 3 has increased or decreased as a same trend, whereas fluctuated in Group 4 and 5. These phenomena might be attributed to that *I*_SAC_ and *I*_Na_Mfb_ enhanced atrial myocytes excitability and triggered spontaneous excitements at large BCLs. [Ca^2+^]_*i*_, the vehicle of EMC, also fluctuated, driving the undulation of mechanical behavior.

Figure 4e–h shows the extremum of four parameters with Mfb–M ratios ranging from 1 to 8. Parameters in Group 1 were constants as no Mfb was coupled to myocytes. Unlike the similar trends of five groups in Fig. 4a–d, the trends of Group 2 and Group 3 in Fig. 4e–h were close and mostly distributed over one side of Group 1, and the trends of Group 4 and Group 5 were similar and distributed over the other side. Our results demonstrated that introducing currents through SACs in myocytes and currents through MGCs in Mfbs in cardiac modeling could lead to different simulation results. In fact, the stretch ability and contractility of myocytes in fibrotic heart were quite different from those in normal heart. Integrated *I*_SAC_ and *I*_MGC_Mfb_ in cardiac simulation could help obtain more accurate and closer to experimental results.

Figure 4i–l shows the extremum of four parameters with *G*_gap_ ranging from 0.5 to 8 nS. Parameters in Group 1 were also constants. The variance of five groups was less than those in other settings (Fig. 4a–h), suggesting the relative small effects of *G*_gap_ on myocyte mechanical parameters. The traces of Group 2 to 5 were mostly distributed over one side of Group 1. Parameters at each *G*_gap_ in Group 4 got the highest or lowest value among five groups, and parameters in Group 5 took the second place, indicating that *I*_SAC_, *I*_Na_Mfb_, and *I*_MGC_Mfb_ played a strong role in atrial myocyte mechanical behavior.

The extremum of *F*_SE_, *F*_PE_, *l*_CE_, *l*_SE_, and *l*_PE_ as functions of BCL, Mfb–M ratio, and *G*_gap_ in isotonic contraction are showed in Fig. 5.

In Fig. 5a–e, the parameters among five groups were close to each other. As BCL increased, the values in Group 1, 4 and 5 first increased and then decreased. In these groups, pure myocyte or integrating *I*_SAC_ and *I*_MGC_Mfb_ in fibrotic myocyte were more likely to cause discordant alternans and mechanical parameters fluctuation at big BCLs.

In Fig. 5f–j, the parameters in Group 2 always stayed over one side of Group 1 as the coupled ratio increased, while the parameters in other groups finally converged over the other side. It suggested that integrating *I*_SAC_, *I*_Na_Mfb_, and *I*_MGC_Mfb_ in fibrotic myocyte significantly influenced the myocyte segment mechanical behavior at large coupled ratios.

In Fig. 5k–o, the parameters in Group 1 were constant, and Group 2, 3, and 5 had the similar traces, while Group 4 got the highest or lowest values. Therefore, *I*_SAC_, *I*_Na_Mfb_, and *I*_MGC_Mfb_ had relative influences on mechanical parameters at large *G*_gap_.

## Discussion

This study investigated the roles of *I*_SAC_, *I*_Na_Mfb_, and *I*_MGC_Mfb_ in myocyte segment mechanical behavior. To address these issues, computational simulations of the coupled Mfb–M system were performed by employing a combination of models of the human atrial myocyte (including *I*_SAC_) and Mfb (including *I*_Na_Mfb_ and *I*_MGC_Mfb_), as well as models of Ca^2+^–force relation and myocyte mechanical segment. Specifically, effects of these currents with changes in (1) BCL, (2) the number of coupled Mfbs, and (3) *G*_gap_ on atrial myocyte segment mechanical parameters were investigated. The integration of *I*_SAC_, *I*_Na_Mfb_, and *I*_MGC_Mfb_ could result in (1) decreased *V*_max_ and APD_90_, increased *V*_rest_ depolarization, and spontaneous excitements even discordant alternans at large BCLs, and (2) increased peak value of *F*_SE_, *F*_segment_, and *l*_SE_ and decreased valley value of *l*_CE_ in isosarcometric contraction, and increased peak value of *F*_SE_ and *l*_SE_ and decreased valley value of *F*_PE_, *l*_CE_, and *l*_PE_ in isotonic contraction. Moreover, *I*_SAC_ and *I*_MGC_Mfb_ have relative effects on myocyte segment mechanical parameters.

### Effects of *I*_SAC_, *I*_Na_Mfb_, and *I*_MGC_Mfb_ on atrial myocyte segment mechanical properties

Effects of *I*_SAC_, *I*_Na_Mfb_, and *I*_MGC_Mfb_ on the excitability of human atrial myocytes have been discussed in our previous study [28]. Here, we discussed the roles of these currents in myocyte segment mechanical behavior.

EMC and MEF were two known effects [7], but the physical role of MEF in EMC was still poorly understood. In general, *I*_SAC_, handling as the major mechanisms of the MEF, was reported to enhance the early phase of AP repolarization and prolong or delay the final phase of repolarization [9, 35, 36]. But the impact of *I*_SAC_ on cardiac mechanics, to our best knowledge, has been rarely studied so far. In our present study, the stretch-activated currents had the most significant influence on myocyte segment mechanical parameters in both isosarcometric contraction and isotonic contraction.

For cardiac Mfbs, many studies have verified that mechanical cues activated cardiac Mfbs and led to increased production of extracellular matrix [37, 38]. Mfbs were regarded as a critical determinant of cardiac mechanics. Previous studies have used computational modeling to demonstrate the acute mechanical effects on cardiac fibroblast structure and organization [39, 40]. They found that an axial strain environment could guide fibroblast proliferation, orientation, and migration [31, 41, 42]. Several groups have simulated cell compaction of collagen gels by calculating mechanical equilibrium between each cell’s contractile forces and nearby collagen fibers’ mechanical properties. They reported that cellular organization is tightly linked to the mechanical feedback loop between cells and matrix [29, 30]. These studies were all about the stretch-induced responses of quiescent cardiac Mfbs. However, the inverse process, i.e., the Mfbs-induced responses of cardiac mechanics, has not been widely explored. Our results showed that coupling Mfbs changed myocytes mechanical properties. In addition, we compared the results of before and after adding *I*_Na_Mfb_ and *I*_MGC_Mfb_ in the Mfb model, and found that the effects of *I*_MGC_Mfb_ on the force of atrial myocytes were contrary to *I*_SAC_.

For *I*_Na_Mfb_, many studies have been conducted to investigate how this current could influence Mfb proliferation [18, 43]. Our results showed that *I*_Na_Mfb_ decreased *V*_max_ and APD_90_ and increased *V*_rest_ depolarization in myocytes. This depolarization changed diastolic Ca^2+^ levels and then altered myocytes mechanical behavior.

For *I*_MGC_Mfb_, experimental data have indicated that cardiac fibroblasts expressed functional MCGs, contributing to the cardiac MEF both under physiological and pathophysiological conditions [44, 45]. We assumed that it could affect myocytes mechanical characteristics like *I*_SAC_. Our results supported this hypothesis. In our simulations, *I*_MGC_Mfb_ altered myocytes mechanical behavior. Interestingly, the effects of *I*_MGC_Mfb_ and *I*_SAC_ on myocyte segment mechanical parameters seemed to be opposite. Myocytes stretch activated *I*_SAC_ and enhanced the influence on mechanical parameters, while Mfbs compression activated *I*_MGC_Mfb_ and weakened the influence. Moreover, MGCs were activated by fibroblast compression and inactivated by fibroblast stretch [21], implying that *I*_MGC_Mfb_ should be integrated in cell modeling only during cell compression, such as fibroblasts/Mfbs compression caused by stretching and dilatation of surrounding cardiac myocytes.

Mfb was a critical determinant of cardiac mechanics. Previous studies have demonstrated that abnormal quantity or organization of Mfb could lead to both systolic and diastolic dysfunction [12, 30, 46]. Besides, previous modeling work suggested that Mfb–M coupling contributed to arrhythmia formation [25, 47]. The key factors included BCL, the number of coupled Mfbs, and *G*_gap_. Here, we integrated *I*_Na_Mfb_, *I*_SAC_, and *I*_MGC_Mfb_ into Mfb–M coupling and compared their effects on myocyte mechanical properties in different settings of BCL, Mfb–M ratio, and *G*_gap_. To the best of our knowledge, this has not been examined before. With BCL, Mfb–M ratio, and *G*_gap_ increasing, impacts of these currents on the extremum of myocyte mechanical parameters became greater, as summarized in Figs. 4 and 5.

## Limitations

Two limitations in the present study should be mentioned. First, functional roles of SACs in Mfbs were not considered. Direct proof of mechanoactivation of mechanosensitive channels in cardiac Mfbs was limited. A handful of experimental studies have found that mechanical cues could lead to the opening of so-called SACs, and transient receptor potential canonical channels were candidates for the stretch-activated currents measured in cardiac fibroblasts [48, 49]. However, the current–voltage relation of *I*_SAC_ in Mfbs needs further study. Second, the breadth of this computational study needs to be extended. Our work focused on the scale of local cell–cell interactions. Other scales, such as scales of subcellular signaling, cell–matrix interactions, tissue remodeling, and organ level conduction properties, were not included in this preliminary study. In fact, processes across these scales did not occur in isolation but operated as an interconnected system with every level passing information to other levels. Therefore, multi-scale modeling frameworks still need to be developed, although they brought computational challenges, and such models involving cardiac Mfbs and fibrosis were still rare.

## Conclusions

This study demonstrated the combinational effects of *I*_SAC_ in myocytes and *I*_Na_Mfb_ and *I*_MGC_Mfb_ in Mfbs on myocyte mechanical properties. Our results showed that the addition of *I*_SAC_, *I*_Na_Mfb_, and *I*_MGC_Mfb_ regulated the peak and valley values of myocyte mechanical parameters in both isosarcometric contraction and isotonic contraction. Effects of these currents on the extremum of myocyte mechanical parameters become more evident as BCL, Mfb–M ratio, and *G*_gap_ increased. The effects proved that the stretch-activated current in atrial myocyte and Na^+^ current and mechano-gated channels in Mfbs should be considered in future pathological cardiac mechanical mathematical modeling, such as atrial fibrillation and cardiac fibrosis.

## Methods

Mathematical model was developed by integrating (1) the model of the human atrial myocyte [50], (2) the model of *I*_SAC_ [33], (3) the model of Ca^2+^–force relation [51, 52], (4) the active model of the human cardiac Mfb [26], (5) our proposed formulation of *I*_Na_Mfb_ and *I*_MGC_Mfb_ based on experimental findings from Chatelier et al. [18] and Kamkin et al. [21], and (6) the Hill three-element rheological scheme of a single segment of myocyte [53, 54]. In following sections, the details of each component of the model will be described.

### The model of Mfb–M coupling

The Mfb–M coupling will be modeled based on [26], with the differential equations for the membrane potential of cardiac Mfb and myocyte are given by

1 $$\frac{{{\text{d}}V_{{{\text{Mfb,}}i}} }}{{{\text{d}}t}} = - \frac{1}{{C_{{m , {\text{Mfb}}}} }}\left( {I_{{{\text{Mfb,}}i}} \left( {V_{{{\text{Mfb,}}i}} ,t} \right) + G_{\text{gap}} \left( {V_{{{\text{Mfb,}}i}} - V_{\text{M}} } \right)} \right)$$

2 $$\frac{{{\text{d}}V_{\text{M}} }}{{{\text{d}}t}} = - \frac{1}{{C_{{m , {\text{M}}}} }}\left( {I_{\text{M}} \left( {V_{\text{M}} ,t} \right) + \mathop \sum \limits_{\text{i = 1}}^{\text{n}} G_{\text{gap}} \left( {V_{\text{M}} - V_{{{\text{Mfb,}}i}} } \right)} \right),$$

where *V*_Mfb,*i*_ and *V*_M_ represent the transmembrane potential of the *i*th coupled Mfb and the human atrial myocyte, *C*_*m*,Mfb_ and *C*_*m*,M_ represent the membrane capacitance of the Mfb and the myocyte, *I*_Mfb,*i*_ and *I*_M_ represent the transmembrane current of the *i*th coupled Mfb and the human atrial myocyte, and *G*_gap_ represents the gap-junctional conductance. It is also noted that a negative *I*_gap_ [i.e., *G*_gap_(*V*_Mfb,*i*_–*V*_M_)] indicates that the current is flowing from the myocyte into the *i*th Mfb, and *n* is the total number of coupled Mfbs.

### Mathematical model of the human atrial myocyte

The mathematical model of the human atrial myocyte developed by Maleckar et al. [50], which is based on experimental data and has correctly replicated APD restitution of the adult human atrial myocyte, was adopted in this study. To examine the influence of the stretch on myocyte AP, the original model from Maleckar et al. is modified with the total ionic current of myocyte (*I*_M_) given as

3 $$\begin{aligned} I_{\text{M}} \left( {V_{\text{M}} ,t} \right) & = I_{\text{Na}} + I_{\text{CaL}} + I_{\text{t}} + I_{\text{Kur}} + I_{\text{K1}} + I_{\text{K,r}} + I_{\text{K,s}} + I_{\text{B,Na}} \\ & \quad + I_{\text{B,Ca}} + I_{\text{NaK}} + I_{\text{CaP}} + I_{\text{NaCa}} + I_{\text{SAC}} - I_{\text{Stim}} , \\ \end{aligned}$$

where *I*_Na_ is fast inward Na^+^ current, *I*_CaL_ L-type Ca^2+^ current, *I*_*t*_ transient outward K^+^ current, *I*_Kur_ sustained outward K^+^ current, *I*_K1_ inward-rectifying K^+^ current, *I*_K,r_ rapid delayed rectifier K^+^ current, *I*_K,s_ slow delayed rectifier K^+^ current, *I*_B,Na_ background Na^+^ current, *I*_B,Ca_ background Ca^2+^ current, *I*_NaK_ Na^+^–K^+^ pump current, *I*_CaP_ sarcolemmal Ca^2+^ pump current, *I*_NaCa_ Na^+^–Ca^2+^ exchange current, *I*_SAC_ stretch-activated current, and *I*_Stim_ stimulated current.

### The model of *I*_SAC_

Kuijpers et al. [55] have conducted experimental studies and reported that *I*_SAC_ in atrial myocytes is permeable to Na^+^, K^+^, and Ca^2+^ [33], and defined as

4 $$I_{\text{SAC}} = I_{\text{SAC,Na}} + I_{\text{SAC,K}} + I_{\text{SAC,Ca}} ,$$

where *I*_SAC,Na_, *I*_SAC,K_, and *I*_SAC,Ca_ represent the contributions of Na^+^, K^+^, and Ca^2+^ to *I*_SAC_, respectively. These currents are defined by the constant-field Goldman–Hodgkin–Katz current equation [56].

To introduce the effect of *I*_SAC_ on intracellular Na^+^, K^+^, and Ca^2+^ concentrations ([Na^+^]_*i*_, [K^+^]_*i*_ and [Ca^2+^]_*i*_), we replace equations of [Na^+^]_*i*_, [K^+^]_*i*_, and [Ca^2+^]_*i*_ in Maleckar et al.’s model [50] as

5 $$\frac{{{\text{d}}\left[ {{\text{Na}}^{ + } } \right]_{i} }}{{{\text{d}}t}} = - \frac{{I_{\text{Na}} + I_{\text{B,Na}} + 3I_{\text{NaK}} + 3I_{\text{NaCa}} + I_{\text{SAC,Na}} }}{{{\text{Vol}}_{i} {\text{F}}}}$$

6 $$\frac{{{\text{d}}\left[ {{\text{K}}^{ + } } \right]_{i} }}{{{\text{d}}t}} = - \frac{{I_{t} + I_{\text{Kur}} + I_{\text{K1}} + I_{{{\text{K,}}s}} + I_{{{\text{K,}}r}} - 2I_{\text{NaK}} + I_{\text{SAC,K}} }}{{{\text{Vol}}_{i} F}}$$

7 $$\frac{{{\text{d}}\left[ {{\text{Ca}}^{ 2+ } } \right]_{i} }}{{{\text{d}}t}} = - \frac{{ - I_{\text{di}} + I_{\text{B,Ca}} + I_{\text{CaP}} - 2I_{\text{NaCa}} + I_{\text{up}} - I_{\text{rel}} + I_{\text{SAC,Ca}} }}{{ 2. 0 {\text{Vol}}_{i} F}} - \frac{{{\text{d}}O}}{{{\text{d}}t}},$$

where *F* is Faraday’s constant, Vol_*i*_ cytosolic volume, *I*_di_ Ca^2+^ diffusion current from the diffusion-restricted subsarcolemmal space to the cytosol, *I*_up_ sarcoplasmic reticulum Ca^2+^ uptake current, *I*_rel_ sarcoplasmic reticulum Ca^2+^ release current, and *O* buffer occupancy.

### The model of the Ca^2+^–force relation

The model 4 of isometric force generation in cardiac myofilaments proposed by Rice et al. was adopted to model the Ca^2+^–force relation [51, 52]. The concentration of Ca^2+^ bound to high-affinity or low-affinity troponin sites is [HTRPNCa] and [LTRPNCa], respectively. The dynamics are governed as

8 $$\frac{{{\text{d}}\left[ {\text{HTRPNCa}} \right]}}{{{\text{d}}t}} = k_{\text{htrpn}}^{ + } \left[ {{\text{Ca}}^{2 + } } \right]_{i} \left( {\left[ {\text{HTRPN}} \right]_{\text{tot}} - \left[ {\text{HTRPNCa}} \right]} \right) - k_{\text{htrpn}}^{ - } \left[ {\text{HTRPNCa}} \right]$$

9 $$\frac{{{\text{d}}\left[ {\text{LTRPNCa}} \right]}}{{{\text{d}}t}} = k_{\text{ltrpn}}^{ + } \left[ {{\text{Ca}}^{2 + } } \right]_{i} \left( {\left[ {\text{LTRPN}} \right]_{\text{tot}} - \left[ {\text{LTRPNCa}} \right]} \right) - k_{\text{ltrpn}}^{ - } \left[ {\text{LTRPNCa}} \right],$$

where [HTRPN]_tot_ represents the total troponin high-affinity site concentration, and $k_{\text{htrpn}}^{ + }$ and $k_{\text{htrpn}}^{ - }$ are the Ca^2+^ on- and off-rates for troponin high-affinity sites. [LTRPN]_tot_ represents the total troponin low-affinity site concentration, and $k_{\text{ltrpn}}^{ + }$ and $k_{\text{ltrpn}}^{ - }$ are the Ca^2+^ on- and off-rates for troponin low-affinity sites.

### The model of human atrial Mfb

The electrophysiological model of human atrial Mfb proposed by MacCannell et al. [26] was used in the present study. It includes time- and voltage-dependent K^+^ current (*I*_Kv_Mfb_), inward-rectifying K^+^ current (*I*_K1_Mfb_), Na^+^–K^+^ pump current (*I*_NaK_Mfb_), and Na^+^ background current (*I*_B,Na_Mfb_).

In addition, *I*_Na_Mfb_ and *I*_MGC_Mfb_ are added in the Mfb model. According to our previous work [28], equations of *I*_Na_Mfb_ and *I*_MGC_Mfb_ are formulated as

10 $$I_{{{\text{Na\_Mfb}}}} = \overline{g}_{\text{Na,Mfb}} m_{\text{Mfb}} j_{\text{Mfb}}^{ 0. 1 2} \left( {V_{\text{Mfb}} - E_{\text{Na,Mfb}} } \right)$$

11 $$E_{\text{Na,Mfb}} = \frac{\text{RT}}{F}{ \log }\frac{{\left[ {{\text{Na}}^{ + } } \right]_{{c , {\text{Mfb}}}} }}{{\left[ {{\text{Na}}^{ + } } \right]_{{i , {\text{Mfb}}}} }}$$

12 $$I_{{{\text{MGC\_Mfb}}}} = \overline{g}_{\text{MGC,Mfb}} \cdot \left( {V_{\text{Mfb}} - E_{\text{MGC,Mfb}} } \right),$$

where $\overline{g}_{\text{Na,Mfb}}$ is the maximum conductance of *I*_Na_Mfb_ (0.756 nS), *E*_Na,Mfb_ the Nernst potential for Na^+^ ions, [Na^+^]_*c*,Mfb_ the Mfb extracellular Na^+^ concentration (130.011 mM), [Na^+^]_*i*,Mfb_ the Mfb intracellular Na^+^ concentration (the initial value is set as 8.5547 mM), and *m*_Mfb_ and *j*_Mfb_ the activation and inactivation parameters, respectively. To follow the experiment data [18], *j* has been modified as *j*^0.12^. $\overline{g}_{\text{MGC,Mfb}}$ is the maximum conductance of *I*_MGC_Mfb_ (0.043 nS), and *E*_MGC,Mfb_ is the reversal potential of MGCs (selected a value close to 0 mV) [21].

### Mechanical behavior of a single segment

The mechanical behavior of a single segment in our model is based on the classical three-element rheological scheme [53, 54].

**Fig. 6:**
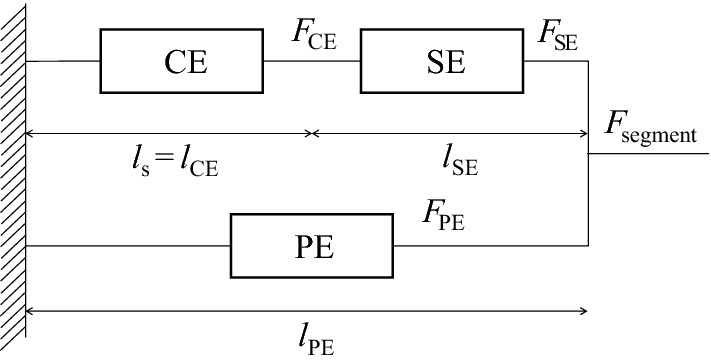
Three-element scheme to model mechanical behavior of a single segment

As shown in Fig. 6, active force (*F*_CE_) is generated by the contractile element (CE), and passive forces (*F*_SE_, *F*_PE_) are generated in a serial elastic element (SE) and a parallel elastic element (PE). *F*_segment_ is the total force generated by the segment. The element lengths are *l*_CE_, *l*_SE_, and *l*_PE_. During mechanical equilibrium, *F*_CE_, *F*_segment_, and *l*_PE_ are defined as

13 $$F_{\text{CE}} = F_{\text{SE}}$$

14 $$F_{\text{segment}} = F_{\text{SE}} + F_{\text{PE}}$$

15 $$l_{\text{PE}} = l_{\text{CE}} + l_{\text{SE}} .$$

### Simulation protocol

We performed single-cell simulations with constant sarcomere length (isosarcometric contraction) and constant applied force (isotonic contraction) to investigate the effects of *I*_SAC_, *I*_Na_Mfb_, and *I*_MGC_Mfb_ on myocyte mechanical properties.

Five groups were simulated sequentially: one atrial myocyte without Mfb coupling (Group 1), one atrial myocyte coupled to two Mfbs without *I*_SAC_, *I*_Na_Mfb_, and *I*_MGC_Mfb_ (Group 2), one atrial myocyte coupled to two Mfbs with *I*_Na_Mfb_ (Group 3), one atrial myocyte coupled to two Mfbs with *I*_SAC_ and *I*_Na_Mfb_ (Group 4), and one atrial myocyte coupled to two Mfbs with *I*_SAC_, *I*_Na_Mfb_, and *I*_MGC_Mfb_ (Group 5).

First, simulations were carried out at a constant *G*_gap_ of 3 nS and a BCL of 1 s. Thereafter, the coupled system was paced with (1) BCLs from 0.1 to 2 s, (2) *G*_gap_ from 0.5 to 8 nS, and (3) number of coupled Mfbs from 1 to 8, to investigate the role of BCL, *G*_gap_, and Mfbs in myocyte mechanical parameters. The maximum or minimum of *F*_SE_, *F*_PE_, *F*_segment_, *l*_CE_, *l*_SE_, and *l*_PE_ at different BCL, *G*_gap_, and Mfbs number were examined.

To ensure the coupled system reached steady-state, stimulation was repeated for 20 cycles. Results from the last cycle in each simulation were used for subsequent analyses. All state variables of the coupled model were updated by means of the forward Euler method. The time step was set to be 10 μs to ensure numerical accuracy and stability. More information on “Methods” is available in the Additional file 1.

## Supplementary information

**Additional file 1.** Additional tables.

## Data Availability

All data generated or analyzed during this study are included in this article.

## Abbreviations

SAC: stretch-activated channel
Mfb: myofibroblast
MGC: mechano-gated channel
Mfb–M: myofibroblast–myocyte
VGSC: voltage-gated sodium channel
AP: action potential
EMC: electromechanical coupling
MEF: mechanoelectrical feedback
TRPM7: transient receptor potential cationic channel subfamily M member 7
APD: duration of the action potential
BCL: basic cycle length
CE: contractile element
SE: serial elastic element
PE: parallel elastic element
