# Supplementary material for: Stretch-activated current in human atrial myocytes and Na+ current and mechano-gated channels’ current in myofibroblasts alter myocyte mechanical behavior: a computational study
Source: Biomed Eng Online. 2019 Oct 25;18:104. doi: 10.1186/s12938-019-0723-5 (PMC6814973; doi:10.1186/s12938-019-0723-5)
Supplement: Supplementary file 1 — Additional file 1. Additional tables. [file 12938_2019_723_MOESM1_ESM.docx]

**Additional Material**

**Model Equations**

Table S1 through 27 contained all the equations, parameters values and initial conditions necessary to carry out the simulations presented in this article. Unless otherwise noted, the units are as follows: time in second (s), voltage in millivolt (mV), concentration in millimolar (mM), current in picoampere (pA), conductance in nanosiemens (nS), capacitance in picofarads (pF), volume in nanoliters (nL), temperature in kelvin (K), and sarcomere length in micron (μm).

**Atrial Myocyte Model**

The atrial myocyte model was represented by the Maleckar et al. mathematical model [[1](#_ENREF_1)], which was based on a previous model of the adult human atrial myocyte action potential (AP), that of Nygren et al. [[2](#_ENREF_2)]. The stimulus used to evoke an AP was a rectangular current pulse (*I*_stim_) with amplitude of 280 pA and duration of 6 ms. Equations of stretch-activated current (*I*_SAC_) were taken from Kuijpers et al [[3](#_ENREF_3)], which was assumed to be a nonselective cation current with a near-linear current-voltage relation on the basis of experimental observations [[4](#_ENREF_4)]. When *I*_SAC_ was integrated in myofibroblast-myocyte (Mfb-M) coupling, equations of intracellular ion concentrations of Na^+^, K^+^ and Ca^2+^ ([Na^+^]_i_, [K^+^]_i_, and [Ca^2+^]_i_) were defined as Table S12. Without *I*_SAC_ in Mfb-M coupling, they were defined as Table S8. The Ca^2+^-force relation was represented by the Rice et al. model 4 [[5](#_ENREF_5)].

**Table S1. Na^+^ current: *I*_Na_**

| $I_{\text{Na}}=P_{\text{Na}}m^{\text{3}}\left( \text{0.9}h_{\text{1}}+\text{0.1}h_{\text{2}} \right){[{Na}^{+}]}_{\text{c}}V_{\text{M}}\frac{\text{F}^{\text{2}}}{\text{RT}}\frac{e^{\left( V_{\text{M}}-E_{\text{Na}} \right)\text{F}/\text{RT}}-\text{1.0}}{e^{V_{\text{M}}\text{F}/\text{RT}}-1.0}$ | |
| --- | --- |
| $\bar{m}=\frac{1.0}{1.0+e^{\left( V_{\text{M}}+\text{27.12} \right)/-\text{8.21}}}$ | $\bar{h}=\frac{1.0}{1.0+e^{\left( V_{\text{M}}+\text{63.6} \right)/5.3}}$ |
| $\frac{dm}{dt}=\frac{\bar{m}-m}{\tau_{m}}$ | $\tau_{m}=0.000042e^{-\left( \left( V_{\text{M}}+\text{25.57} \right)/\text{28.8} \right)^{\text{2}}}+0.000024$ |
| $\frac{dh_{1}}{dt}=\frac{\bar{h}-h_{\text{1}}}{\tau_{h_{\text{1}}}}$ | $\tau_{h_{\text{1}}}=\frac{0.03}{1.0+e^{\left( V_{\text{M}}+\text{35.1} \right)/\text{3.2}}}+0.0003$ |
| $\frac{dh_{2}}{dt}=\frac{\bar{h}-h_{\text{2}}}{\tau_{h_{\text{2}}}}$ | $\tau_{h_{\text{2}}}=\frac{0.12}{1.0+e^{\left( V_{\text{M}}+\text{35.1} \right)/\text{3.2}}}+0.003$ |
| $E_{\text{Na}}=\frac{\text{RT}}{\text{F}}\text{log}\frac{{[\text{Na}^{+}]}_{\text{c}}}{{[\text{Na}^{+}]}_{\text{i}}}$ |  |

**Table S2. Ca^2+^ current: *I*_CaL_**

| $I_{\text{CaL}}=\bar{g}_{\text{C}\text{a}\text{L}}d_{\text{L}}\left[ f_{\text{Ca}}f_{\text{L1}}+\left( 1-f_{\text{Ca}} \right)f_{\text{L2}} \right]\left( V_{\text{M}}-E_{\text{Ca,app}} \right)$ | |
| --- | --- |
| $\bar{d}_{\text{L}}=\frac{1.0}{1.0+e^{\left( V_{\text{M}}+\text{9.0} \right)/\text{-}\text{5.8}}}$ | $\bar{f}_{\text{L}}=\frac{1.0}{1.0+e^{\left( V_{\text{M}}+\text{27.4} \right)/\text{7.1}}}$ |
| $\frac{dd_{\text{L}}}{dt}=\frac{\bar{d}_{\text{L}}-d_{\text{L}}}{\tau_{d_{\text{L}}}}$ | $\tau_{d_{\text{L}}}=0.0027e^{-\left( \left( V_{\text{M}}+\text{35.0} \right)/\text{30.0} \right)^{\text{2}}}+0.002$ |
| $\frac{df_{\text{L}1}}{dt}=\frac{\bar{f}_{\text{L}}-f_{\text{L}1}}{\tau_{f_{\text{L}1}}}$ | $\tau_{f_{\text{L}1}}=0.161e^{-\left( \left( V_{\text{M}}+\text{40.0} \right)/\text{14.4} \right)^{\text{2}}}+0.01$ |
| $\frac{df_{\text{L}2}}{dt}=\frac{\bar{f}_{\text{L}}-f_{\text{L}2}}{\tau_{f_{\text{L}2}}}$ | $\tau_{f_{\text{L}\text{2}}}=1.3323e^{-\left( \left( V_{\text{M}}+\text{40.0} \right)/\text{14.2} \right)^{\text{2}}}+0.0626$ |
| $f_{\text{Ca}}=\frac{\left[ \text{Ca}^{\text{2+}} \right]_{\text{d}}}{\left[ \text{Ca}^{\text{2+}} \right]_{\text{d}}+k_{\text{Ca}}}$ |  |

**Table S3. Transient and ultrarapidly delayed rectifier K^+^ currents: *I*_t_ and *I*_Kur_**

| $I_{\text{t}}=\bar{g}_{\text{t}}rs\left( V_{\text{M}}-E_{\text{K}} \right)$ | |
| --- | --- |
| $\bar{r}=\frac{1.0}{1.0+e^{\left( V_{\text{M}}-\text{1.0} \right)/\text{-}\text{11.0}}}$ | $\bar{s}=\frac{1.0}{1.0+e^{\left( V_{\text{M}}+\text{40.5} \right)/\text{11.5}}}$ |
| $\frac{dr}{dt}=\frac{\bar{r}-r}{\tau_{r}}$ | $\tau_{r}=0.0035e^{-\left( V_{\text{M}}/\text{30.0} \right)^{\text{2}}}+0.0015$ |
| $\frac{ds}{dt}=\frac{\bar{s}-s}{\tau_{s}}$ | $\tau_{s}=0.025635e^{-\left( \left( V_{\text{M}}+\text{52.45} \right)/\text{1}\text{5.89} \right)^{\text{2}}}+0.01414$ |
| $I_{\text{Kur}}=\bar{g}_{\text{Kur}}r_{\text{Kur}}s_{\text{Kur}}\left( V_{\text{M}}-E_{\text{K}} \right)$ | |
| $\bar{r}_{\text{Kur}}=\frac{1.0}{1.0+e^{\left( V_{\text{M}}+\text{6.0} \right)/\text{-}\text{8.6}}}$ | $\bar{s}_{\text{Kur}}=\frac{1.0}{1.0+e^{\left( V_{\text{M}}+\text{7.5} \right)/\text{10.0}}}$ |
| $\frac{dr_{\text{Kur}}}{dt}=\frac{\bar{r}_{\text{Kur}}-r_{\text{Kur}}}{\tau_{r_{\text{Kur}}}}$ | $\tau_{r_{\text{Kur}}}=\frac{0.009}{1.0+e^{\left( V_{\text{M}}+\text{5.0} \right)/\text{12.0}}}+0.0005$ |
| $\frac{ds_{\text{Kur}}}{dt}=\frac{\bar{s}_{\text{Kur}}-s_{\text{Kur}}}{\tau_{s_{\text{Kur}}}}$ | $\tau_{s_{\text{Kur}}}=\frac{0.59}{1.0+e^{\left( V_{\text{M}}+\text{60.0} \right)/\text{10.0}}}+3.05$ |
| $E_{\text{K}}=\frac{\text{RT}}{\text{F}}\text{log}\frac{{[\text{K}^{+}]}_{\text{c}}}{{[\text{K}^{+}]}_{\text{i}}}$ |  |

**Table S4. Delayed rectifier K^+^ currents: *I*_K,s_ and *I*_K,r_**

| $I_{\text{K,s}}=\bar{g}_{\text{K,s}}n\left( V_{\text{M}}-E_{\text{K}} \right)$ | |
| --- | --- |
| $\bar{n}=\frac{1.0}{1.0+e^{\left( V_{\text{M}}-\text{1}\text{9.9} \right)/\text{-}\text{12.7}}}$ | $\tau_{n}=0.7+0.4e^{-\left( \left( V_{\text{M}}-\text{20.0} \right)/\text{20.0} \right)^{\text{2}}}$ |
| $\frac{dn}{dt}=\frac{\bar{n}-n}{\tau_{n}}$ | $E_{\text{K}}=\frac{\text{RT}}{\text{F}}\text{log}\frac{{[\text{K}^{+}]}_{\text{c}}}{{[\text{K}^{+}]}_{\text{i}}}$ |
| $I_{\text{K,r}}=\bar{g}_{\text{K,r}}p_{\text{a}}p_{\text{i}}\left( V_{\text{M}}-E_{\text{K}} \right)$ | |
| $\bar{p}_{\text{a}}=\frac{1.0}{1.0+e^{\left( V_{\text{M}}+\text{15.0} \right)/\text{-}\text{6.0}}}$ | $p_{\text{i}}=\frac{1.0}{1.0+e^{\left( V_{\text{M}}+\text{55.0} \right)/\text{24}\text{.0}}}$ |
| $\frac{dp_{\text{a}}}{dt}=\frac{\bar{p}_{\text{a}}-p_{\text{a}}}{\tau_{p_{\text{a}}}}$ | $\tau_{p_{\text{a}}}=0.03118+0.21718e^{-\left( \left( V_{\text{M}}+\text{20.1376} \right)/\text{22.1996} \right)^{\text{2}}}$ |

**Table S5. Inward rectifier K^+^ currents: *I*_K1_**

| $I_{\text{K1}}=\bar{g}_{\text{K1}}{[\text{K}^{+}]}_{\text{c}}^{\text{0.4457}}\frac{V_{\text{M}}-E_{\text{K}}}{1.0+e^{1.5\left( V_{\text{M}}-E_{\text{K}}+\text{3.6} \right)\text{F}/\text{RT}}}$ |
| --- |
| $E_{\text{K}}=\frac{\text{RT}}{\text{F}}\text{log}\frac{{[\text{K}^{+}]}_{\text{c}}}{{[\text{K}^{+}]}_{\text{i}}}$ |

**Table S6. Background inward currents: *I*_B,Na_ and *I*_B,Ca_**

| $I_{\text{B,Na}}=\bar{g}_{\text{B,Na}}\left( V_{\text{M}}-E_{\text{Na}} \right)$ | $I_{\text{B,Ca}}=\bar{g}_{\text{B,Ca}}\left( V_{\text{M}}-E_{\text{C}\text{a}} \right)$ |
| --- | --- |
| $E_{\text{Na}}=\frac{\text{RT}}{\text{F}}\text{log}\frac{{[\text{Na}^{+}]}_{\text{c}}}{{[\text{Na}^{+}]}_{\text{i}}}$ | $E_{\text{Ca}}=\frac{\text{RT}}{\text{2}\text{F}}\text{log}\frac{{[{\text{C}\text{a}}^{\text{2+}}]}_{\text{c}}}{{[{\text{C}\text{a}}^{\text{2+}}]}_{\text{i}}}$ |

**Table S7. Pump and exchanger currents: *I*_NaK_, *I*_CaP_, and *I*_NaCa_**

| $I_{\text{NaK}}=\bar{I}_{\text{NaK}}\frac{{[\text{K}^{+}]}_{\text{c}}}{{[\text{K}^{+}]}_{\text{c}}+k_{\text{NaK,K}}}\cdot\frac{{[\text{Na}^{+}]}_{\text{i}}^{\text{1.5}}}{{[\text{Na}^{+}]}_{\text{i}}^{\text{1.5}}+k_{\text{NaK,Na}}^{\text{1.5}}}\cdot\frac{V_{\text{M}}+150.0}{V_{\text{M}}+200.0}$ |
| --- |
| $I_{\text{CaP}}=\bar{I}_{\text{CaP}}\frac{{[\text{Ca}^{\text{2+}}]}_{\text{i}}}{{[\text{Ca}^{\text{2+}}]}_{\text{i}}+k_{\text{CaP}}}$ |
| $I_{\text{NaCa}}=k_{\text{NaCa}}\frac{{[\text{Na}^{+}]}_{\text{i}}^{\text{3}}{[\text{Ca}^{\text{2+}}]}_{\text{c}}e^{\text{γVF/RT}}-{[\text{Na}^{+}]}_{\text{c}}^{\text{3}}{[\text{Ca}^{\text{2+}}]}_{\text{i}}e^{\left( \text{γ}\text{-1.0} \right)\text{VF/RT}}}{1.0+d_{\text{NaCa}}\left( {[\text{Na}^{+}]}_{\text{c}}^{\text{3}}{[\text{Ca}^{\text{2+}}]}_{\text{i}}+{[\text{Na}^{+}]}_{\text{i}}^{\text{3}}{[\text{Ca}^{\text{2+}}]}_{\text{c}} \right)}$ |

**Table S8. Intracellular ion concentrations: [Na^+^]_i_, [K^+^]_i_, and [Ca^2+^]_i_**

| $\frac{{d\left[ \text{Na}^{+} \right]}_{\text{i}}}{dt}=-\frac{I_{\text{Na}}+I_{\text{B,Na}}+{3I}_{\text{NaK}}+{3I}_{\text{NaCa}}}{\text{Vol}_{\text{i}}\text{F}}$ |
| --- |
| $\frac{{d\left[ \text{K}^{+} \right]}_{\text{i}}}{dt}=-\frac{I_{\text{t}}+I_{\text{Kur}}+I_{\text{K1}}+I_{\text{K,s}}+I_{\text{K,r}}-2I_{\text{NaK}}}{\text{Vol}_{\text{i}}\text{F}}$ |
| $\frac{{d\left[ \text{Ca}^{\text{2}+} \right]}_{\text{i}}}{dt}=-\frac{{-I}_{\text{di}}+I_{\text{B,Ca}}+I_{\text{CaP}}-2I_{\text{NaCa}}+I_{\text{up}}-I_{\text{rel}}}{{\text{2.0 }\text{Vol}}_{\text{i}}\text{F}}-\frac{dO}{dt}$ |
| $\frac{dO}{dt}=0.08\frac{dO_{\text{TC}}}{dt}+0.16\frac{dO_{\text{T}\text{Mg}\text{C}}}{dt}+0.045\frac{dO_{\text{C}}}{dt}$ |
| $\frac{{d\left[ \text{Ca}^{\text{2}+} \right]}_{\text{d}}}{dt}=-\frac{I_{\text{CaL}}{-I}_{\text{di}}}{{\text{2.0 }\text{Vol}}_{\text{d}}\text{F}}$ |
| $I_{\text{di}}=\left( \left[ \text{Ca}^{\text{2}+} \right]_{\text{d}}-\left[ \text{Ca}^{\text{2}+} \right]_{\text{i}} \right)\frac{2\text{F}{\text{ }\text{Vol}}_{\text{d}}}{\tau_{\text{di}}}$ |

**Table S9. Cleft space ion concentrations: [Na^+^]_c_, [K^+^]_c_, and [Ca^2+^]_c_**

| $\frac{{d\left[ \text{Na}^{+} \right]}_{\text{c}}}{dt}=\frac{\left[ \text{Na}^{+} \right]_{\text{b}}-\left[ \text{Na}^{+} \right]_{\text{c}}}{\tau_{\text{Na}}}+\frac{I_{\text{Na}}+I_{\text{B,Na}}+{3I}_{\text{NaK}}+{3I}_{\text{NaCa}}}{\text{Vol}_{\text{c}}\text{F}}$ |
| --- |
| $\frac{{d\left[ \text{K}^{+} \right]}_{\text{c}}}{dt}=\frac{\left[ \text{K}^{+} \right]_{\text{b}}-\left[ \text{K}^{+} \right]_{\text{c}}}{\tau_{\text{K}}}+\frac{I_{\text{t}}+I_{\text{Kur}}+I_{\text{K1}}+I_{\text{K,s}}+I_{\text{K,r}}-2I_{\text{NaK}}}{\text{Vol}_{\text{c}}\text{F}}$ |
| $\frac{{d\left[ \text{Ca}^{\text{2}+} \right]}_{\text{c}}}{dt}=\frac{\left[ \text{Ca}^{\text{2}+} \right]_{\text{b}}-\left[ \text{Ca}^{\text{2}+} \right]_{\text{c}}}{\tau_{\text{Ca}}}+\frac{I_{\text{CaL}}+I_{\text{B,Ca}}+I_{\text{CaP}}-2I_{\text{NaCa}}}{{\text{2.0 }\text{Vol}}_{\text{c}}\text{F}}$ |

**Table S10. Intracellular Ca^2+^ buffering**

| $\frac{{d\text{O}}_{\text{C}}}{dt}=\text{200000.0}\left[ \text{Ca}^{\text{2}+} \right]_{\text{i}}\left( 1.0-O_{\text{C}} \right)-476.0O_{\text{C}}$ |
| --- |
| $\frac{{d\text{O}}_{\text{TC}}}{dt}=78400.0\left[ \text{Ca}^{\text{2}+} \right]_{\text{i}}\left( 1.0-O_{\text{T}\text{C}} \right)-392.0O_{\text{T}\text{C}}$ |
| $\frac{{d\text{O}}_{\text{TMgC}}}{dt}=200000.0\left[ \text{Ca}^{\text{2}+} \right]_{\text{i}}\left( 1.0-O_{\text{TMg}\text{C}}-O_{\text{TMgMg}} \right)-6.6O_{\text{TMgC}}$ |
| $\frac{{d\text{O}}_{\text{TMgMg}}}{dt}=2000.0\left[ \text{Mg}^{\text{2}+} \right]_{\text{i}}\left( 1.0-O_{\text{TMg}\text{C}}-O_{\text{TMgMg}} \right)-666.0O_{\text{TMgMg}}$ |

**Table S11. Ca^2+^ handling by the sarcoplasmic reticulum**

| $I_{\text{up}}=\bar{I}_{\text{up}}\frac{\left[ \text{Ca}^{\text{2}+} \right]_{\text{i}}/k_{\text{cyca}}-k_{\text{xcs}}^{\text{2}}\left[ \text{Ca}^{\text{2}+} \right]_{\text{up}}/k_{\text{sr}\text{ca}}}{\left( \left[ \text{Ca}^{\text{2}+} \right]_{\text{i}}+k_{\text{cyca}} \right)/k_{\text{cyca}}+k_{\text{xcs}}\left( \left[ \text{Ca}^{\text{2}+} \right]_{\text{up}}+k_{\text{sr}\text{ca}} \right)/k_{\text{sr}\text{ca}}}$ |
| --- |
| $I_{\text{tr}}=\left( \left[ \text{Ca}^{\text{2}+} \right]_{\text{up}}-\left[ \text{Ca}^{\text{2}+} \right]_{\text{rel}} \right)\frac{2\text{F}{\text{ }\text{Vol}}_{\text{rel}}}{\tau_{\text{tr}}}$ |
| $I_{\text{rel}}=\alpha_{\text{rel}}\left( \frac{F_{\text{2}}}{F_{\text{2}}+0.25} \right)^{2}\left( \left[ \text{Ca}^{\text{2}+} \right]_{\text{rel}}-\left[ \text{Ca}^{\text{2}+} \right]_{\text{i}} \right)$ |
| $\frac{{d\text{O}}_{\text{Calse}}}{dt}=480.0\left[ \text{Ca}^{\text{2}+} \right]_{\text{rel}}\left( 1.0-O_{\text{Calse}} \right)-400.0O_{\text{Calse}}$ |
| $\frac{{d\left[ \text{Ca}^{\text{2}+} \right]}_{\text{rel}}}{dt}=\frac{I_{\text{tr}}-I_{\text{rel}}}{2\text{F}{\text{ }\text{Vol}}_{\text{rel}}}-31.0\frac{{d\text{O}}_{\text{Calse}}}{dt}$ |
| $\frac{{d\left[ \text{Ca}^{\text{2}+} \right]}_{\text{up}}}{dt}=\frac{I_{\text{up}}-I_{\text{tr}}}{2\text{F}{\text{ }\text{Vol}}_{\text{up}}}$ |
| $\frac{{d\text{F}}_{\text{1}}}{dt}=\text{r}_{\text{recov}}\left( 1.0-\text{F}_{\text{1}}-\text{F}_{\text{2}} \right)-r_{\text{act}}\text{F}_{\text{1}}$ |
| $\frac{{d\text{F}}_{\text{2}}}{dt}=r_{\text{act}}\text{F}_{\text{1}}-r_{\text{in}\text{act}}\text{F}_{\text{2}}$ |
| $r_{\text{act}}=203.8\left[ \left( \frac{\left[ \text{Ca}^{\text{2}+} \right]_{\text{i}}}{\left[ \text{Ca}^{\text{2}+} \right]_{\text{i}}+k_{\text{rel,i}}} \right)^{4}+\left( \frac{\left[ \text{Ca}^{\text{2}+} \right]_{\text{d}}}{\left[ \text{Ca}^{\text{2}+} \right]_{\text{d}}+k_{\text{rel,d}}} \right)^{4} \right]$ |
| $r_{\text{in}\text{act}}=33.96+339.6\left( \frac{\left[ \text{Ca}^{\text{2}+} \right]_{\text{i}}}{\left[ \text{Ca}^{\text{2}+} \right]_{\text{i}}+k_{\text{rel,i}}} \right)^{4}$ |

**Table S12. Stretch-activated current: *I*_SAC_**

| $I_{\text{SAC}}=I_{\text{SAC,Na}}+I_{\text{SAC,K}}+I_{\text{SAC,Ca}}$ |
| --- |
| $I_{\text{SAC,Na}}=\bar{P}_{\text{Na}}g_{\text{SAC}}\frac{\text{z}_{\text{Na}}^{\text{2}}\text{F}^{\text{2}}V_{\text{M}}}{\text{RT}}\cdot\frac{\left[ \text{Na}^{+} \right]_{\text{i}}-\left[ \text{Na}^{+} \right]_{\text{c}}e^{-\left( z_{\text{Na}}\text{F}V_{\text{M}} \right)/\text{RT}}}{1.0-e^{-\left( z_{\text{Na}}\text{F}V_{\text{M}} \right)/\text{RT}}}$ |
| $I_{\text{SAC,K}}=\bar{P}_{\text{K}}g_{\text{SAC}}\frac{\text{z}_{\text{K}}^{\text{2}}\text{F}^{\text{2}}V_{\text{M}}}{\text{RT}}\cdot\frac{\left[ \text{K}^{+} \right]_{\text{i}}-\left[ \text{K}^{+} \right]_{\text{c}}e^{-\left( z_{\text{K}}\text{F}V_{\text{M}} \right)/\text{RT}}}{1.0-e^{-\left( z_{\text{K}}\text{F}V_{\text{M}} \right)/\text{RT}}}$ |
| $I_{\text{SAC,Ca}}=\bar{P}_{\text{C}\text{a}}g_{\text{SAC}}\frac{\text{z}_{\text{C}\text{a}}^{\text{2}}\text{F}^{\text{2}}V_{\text{M}}}{\text{RT}}\cdot\frac{\left[ \text{Ca}^{\text{2}+} \right]_{\text{i}}-\left[ \text{Ca}^{\text{2}+} \right]_{\text{c}}e^{-\left( z_{\text{C}\text{a}}\text{F}V_{\text{M}} \right)/\text{RT}}}{1.0-e^{-\left( z_{\text{C}\text{a}}\text{F}V_{\text{M}} \right)/\text{RT}}}$ |
| $g_{\text{SAC}}=\frac{G_{\text{SAC}}}{1.0+K_{\text{SAC}}e^{-\left( \alpha_{\text{SAC}}\left( -\text{1} \right) \right)}}$ |
| $\bar{P}_{\text{Na}}:\bar{P}_{\text{K}}:\bar{P}_{\text{C}\text{a}}=\text{1}:\text{1}:\text{1}$ |
| $\frac{{d\left[ \text{Na}^{+} \right]}_{\text{i}}}{dt}=-\frac{I_{\text{Na}}+I_{\text{B,Na}}+{3I}_{\text{NaK}}+{3I}_{\text{NaCa}}+I_{\text{SAC,Na}}}{\text{Vol}_{\text{i}}\text{F}}$ |
| $\frac{{d\left[ \text{K}^{+} \right]}_{\text{i}}}{dt}=-\frac{I_{\text{t}}+I_{\text{Kur}}+I_{\text{K1}}+I_{\text{K,s}}+I_{\text{K,r}}-2I_{\text{NaK}}+I_{\text{SAC,K}}}{\text{Vol}_{\text{i}}\text{F}}$ |
| $\frac{{d\left[ \text{Ca}^{\text{2}+} \right]}_{\text{i}}}{dt}=-\frac{{-I}_{\text{di}}+I_{\text{B,Ca}}+I_{\text{CaP}}-2I_{\text{NaCa}}+I_{\text{up}}-I_{\text{rel}}+I_{\text{SAC,Ca}}}{{\text{2.0 }\text{Vol}}_{\text{i}}\text{F}}-\frac{dO}{dt}$ |

**Table S13. Myofilaments troponin**

| $\frac{d\left[ \mathrm{HTRPNCa} \right]}{dt}=k_{\mathrm{htrpn}}^{+}\left[ \mathrm{Ca}^{2+} \right]_{i}\left( \left[ \mathrm{HTRPN} \right]_{\mathrm{tot}}-\left[ \mathrm{HTRPNCa} \right] \right)-k_{\mathrm{htrpn}}^{-}\left[ \mathrm{HTRPNCa} \right]$ |
| --- |
| $\frac{d\left[ \mathrm{LTRPNCa} \right]}{dt}=k_{\mathrm{ltrpn}}^{+}\left[ \mathrm{Ca}^{2+} \right]_{i}\left( \left[ \mathrm{LTRPN} \right]_{\mathrm{tot}}-\left[ \mathrm{LTRPNCa} \right] \right)-k_{\mathrm{ltrpn}}^{-}\left[ \mathrm{LTRPNCa} \right]$ |

**Table S14. Tropomyosin/cross bridges**

| $f_{01}=3\cdot f_{\mathrm{XB}}$ | $g_{01}(SL)=1\cdot g_{\mathrm{XB}}(SL)$ |
| --- | --- |
| $f_{12}=10\cdot f_{\mathrm{XB}}$ | $g_{12}(SL)=2\cdot g_{\mathrm{XB}}(SL)$ |
| $f_{23}=7\cdot f_{\mathrm{XB}}$ | $g_{23}(SL)=3\cdot g_{\mathrm{XB}}(SL)$ |
| $\mathrm{SL}_{\mathrm{norm}}=\left( \mathrm{SL}-1.7 \right)/0.6$ | $g_{\mathrm{XB}}(SL)=g_{\mathrm{XB}}^{*}\left[ {1+\left( 1-\mathrm{SL}_{\mathrm{norm}} \right)}^{1.6} \right]$ |
| $k_{\mathrm{np}}^{\mathrm{trop}}=k_{\mathrm{pn}}^{\mathrm{trop}}\cdot\left\{ \frac{\left[ \mathrm{LTRPNCa} \right]/\left[ \mathrm{LTRPN} \right]_{\mathrm{tot}}}{K_{0.5}^{\mathrm{trop}}} \right\}^{N^{\mathrm{trop}}}$ | $N^{\mathrm{trop}}=3.5+2.5\cdot\mathrm{SL}_{\mathrm{norm}}$ |
| $K_{0.5}^{\mathrm{trop}}=\left( 1+\frac{K_{\mathrm{Ca}}^{\mathrm{trop}}}{1.7-0.9\cdot\mathrm{SL}_{\mathrm{norm}}} \right)^{-1}$ | $K_{\mathrm{Ca}}^{\mathrm{trop}}=k_{\mathrm{ltrpn}}^{-}/k_{\mathrm{ltrpn}}^{+}$ |
| $\frac{dN0}{dt}=k_{\mathrm{pn}}^{\mathrm{trop}}P0-k_{\mathrm{np}}^{\mathrm{trop}}N0+g_{01}(SL)\cdot N1$ | |
| $\frac{dP0}{dt}=-\left( k_{\mathrm{pn}}^{\mathrm{trop}}+f_{01} \right)P0+k_{\mathrm{np}}^{\mathrm{trop}}N0+g_{01}(SL)\cdot P1$ | |
| $\frac{dP1}{dt}=-\left[ k_{\mathrm{pn}}^{\mathrm{trop}}+f_{12}+g_{01}\left( \mathrm{SL} \right) \right]P1+k_{\mathrm{np}}^{\mathrm{trop}}N1+f_{01}\cdot P0+g_{12}(SL)\cdot P2$ | |
| $\frac{dP2}{dt}=-\left[ f_{23}+g_{12}\left( \mathrm{SL} \right) \right]P2+f_{12}P1+g_{23}(SL)\cdot P3$ | |
| $\frac{dP3}{dt}={-g_{23}\left( \mathrm{SL} \right)\cdot P3+f}_{23}P2$  $\frac{dN1}{dt}=k_{\mathrm{pn}}^{\mathrm{trop}}P1-\left[ k_{\mathrm{np}}^{\mathrm{trop}}+g_{01}(SL) \right]\cdot N1$ | |

**Table S15. Force computation**

| $Force=\zeta\cdot F_{\mathrm{norm}}$ |
| --- |
| $F_{\mathrm{norm}}=\phi\left( \mathrm{SL} \right)\cdot\frac{P1+N1+2P2+3P3}{{Force}_{\max}}$ |
| $\phi\left( \mathrm{SL} \right)=\left\{ \begin{aligned} \left( \mathrm{SL}-0.6 \right)/1.4,\mathrm{if} 1.7\mu m\leq\mathrm{SL}\leq2.0\mu m \\ 1,\mathrm{if} 2.0\mu m<\mathrm{SL}\leq2.2\mu m \\ \left( 3.6-SL \right)/1.4,\mathrm{if} 2.2\mu m<\mathrm{SL}\leq2.3\mu m \end{aligned} \right.$ |
| ${Force}_{\max}={P1}_{\max}+2{P2}_{\max}+3{P3}_{\max}$ |
| ${P1}_{\max}=\frac{f_{01}\cdot2g_{\mathrm{XB}}^{*}\cdot3g_{\mathrm{XB}}^{*}}{\sum\mathrm{paths}}$ |
| ${P2}_{\max}=\frac{f_{01}\cdot f_{12}\cdot3g_{\mathrm{XB}}^{*}}{\sum\mathrm{paths}}$ |
| ${P3}_{\max}=\frac{f_{01}\cdot f_{12}\cdot f_{23}}{\sum\mathrm{paths}}$ |
| $\sum\mathrm{paths}=g_{\mathrm{XB}}^{*}\cdot2g_{\mathrm{XB}}^{*}\cdot3g_{\mathrm{XB}}^{*}+f_{01}\cdot2g_{\mathrm{XB}}^{*}\cdot3g_{\mathrm{XB}}^{*}+f_{01}\cdot f_{12}\cdot3g_{\mathrm{XB}}^{*}+f_{01}\cdot f_{12}\cdot f_{23}$ |

**Atrial Myofibroblast Model**

The atrial Mfb model was represented by the MacCannell et al. mathematical model [[6](#_ENREF_6)]. Mathematical formulations of the currents through voltage-gated sodium channels (*I*_Na_Mfb_) and mechano-gated channels (*I*_MGC_Mfb_) was based upon experimental results from Chatelier et al. [[7](#_ENREF_7)] and Kamkin et al. [[8](#_ENREF_8)], respectively.

**Table S16. Time- and voltage-dependent K^+^ current: I_Kv_Mfb_**

| $I_{\text{Kv}\text{\_Mfb}}=\bar{g}_{\text{Kv}\text{,Mfb}}r_{\text{Kv}}s_{\text{Kv}}\left( V_{\text{Mfb}}-E_{\text{K}\text{,Mfb}} \right)$ | |
| --- | --- |
| $\bar{r}_{\text{Kv}}=\left( 1.0+e^{-\left( V_{\text{Mfb}}+\text{20.0} \right)/\text{11.0}} \right)^{\text{-1}}$ | $\bar{s}_{\text{Kv}}=\left( 1.0+e^{\left( V_{\text{Mfb}}+\text{23.0} \right)/\text{7.0}} \right)^{\text{-1}}$ |
| $\frac{dr_{\text{Kv}}}{dt}=\frac{\bar{r}_{\text{Kv}}-r_{\text{Kv}}}{\tau_{r_{\text{Kv}}}}$ | $\tau_{r_{\text{Kv}}}=0.0203+0.138\cdot e^{\left( -\left( V_{\text{Mfb}}+\text{20.0} \right)/\text{25.9} \right)^{\text{2}}}$ |
| $\frac{ds_{\text{Kv}}}{dt}=\frac{\bar{s}_{\text{Kv}}-s_{\text{Kv}}}{\tau_{s_{\text{Kv}}}}$ | $\tau_{s_{\text{Kv}}}=1.574+5.268\cdot e^{\left( -\left( V_{\text{Mfb}}+\text{23.0} \right)/\text{22.7} \right)^{\text{2}}}$ |
| $E_{\text{K}\text{,Mfb}}=\frac{\text{RT}}{\text{F}}\text{log}\frac{{[\text{K}^{+}]}_{\text{c,Mfb}}}{{[\text{K}^{+}]}_{\text{i,Mfb}}}$ | |

**Table S17. Time-independent inward-rectifying K+ current: *I*_K1_Mfb_**

| $I_{\text{K1}\text{\_Mfb}}=\bar{g}_{\text{K1}\text{,Mfb}}\left( \alpha_{\text{K1}}/\left( \alpha_{\text{K1}}+\beta_{\text{K1}} \right) \right)\left( V_{\text{Mfb}}-E_{\text{K}\text{,Mfb}} \right)$ |
| --- |
| $\alpha_{\text{K1}}=\text{0.1}\left( \text{1.0}+e^{\text{0.06}\left( V_{\text{Mfb}}-E_{\text{K}}-\text{200.0} \right)} \right)^{\text{-1}}$ |
| $\beta_{\text{K1}}=\frac{3.0\cdot e^{\text{0.0002}\left( V_{\text{Mfb}}-E_{\text{K}}+\text{100.0} \right)}+e^{\text{0.}\text{1}\left( V_{\text{Mfb}}-E_{\text{K}}+\text{10.0} \right)}}{1.0+e^{-\text{0.5}\left( V_{\text{Mfb}}-E_{\text{K}} \right)}}$ |
| $E_{\text{K}\text{,Mfb}}=\frac{\text{RT}}{\text{F}}\text{log}\frac{{[\text{K}^{+}]}_{\text{c,Mfb}}}{{[\text{K}^{+}]}_{\text{i,Mfb}}}$ |

**Table S18. Na^+^-K^+^ pump current: *I*_NaK_Mfb_**

| $I_{\text{NaK}\text{\_Mfb}}=\bar{I}_{\text{NaK}\text{,Mfb}}\cdot\frac{{[\text{K}^{+}]}_{\text{c,Mfb}}}{{[\text{K}^{+}]}_{\text{c,Mfb}}+k_{\text{mK}}}\cdot\frac{{{[\text{Na}^{+}]}_{\text{i,Mfb}}}^{\text{1.5}}}{{{[\text{Na}^{+}]}_{\text{i,Mfb}}}^{\text{1.5}}+{k_{\text{mNa}}}^{\text{1.5}}}\cdot\frac{V_{\text{Mfb}}+150.0}{V_{\text{Mfb}}+\text{200.0}}$ |
| --- |

**Table S19. Background inward current: *I*_B,Na_Mfb_**

| $I_{\text{B,Na\_Mfb}}=\bar{g}_{\text{B,Na,Mfb}}\left( V_{\text{M}\text{fb}}-E_{\text{Na}\text{,Mfb}} \right)$ |
| --- |
| $E_{\text{Na,Mfb}}=\frac{\text{RT}}{\text{F}}\text{log}\frac{{[\text{Na}^{+}]}_{\text{c,Mfb}}}{{[\text{Na}^{+}]}_{\text{i,Mfb}}}$ |

**Table S20. Na^+^ current: *I*_Na_Mfb_**

| $I_{\text{Na}\text{\_Mfb}}=\bar{g}_{\text{Na}\text{,Mfb}}m_{\text{Mfb}}j_{\text{Mfb}}^{\text{0.12}}\left( V_{\text{M}\text{fb}}-E_{\text{Na}\text{,Mfb}} \right)$ | |
| --- | --- |
| $\bar{m}_{\text{Mfb}}=\frac{-0.0102-1.0063}{1.0+e^{\left( V_{\text{M}\text{fb}}+42.1 \right)/10.53}}+1.0063$ | $\bar{j}_{\text{Mfb}}=\frac{1.04-0.004}{1.0+e^{\left( V_{\text{M}\text{fb}}+84.82 \right)/9.4}}+0.004$ |
| $\frac{dm_{\text{Mfb}}}{dt}=\frac{\bar{m}_{\text{Mfb}}-m_{\text{Mfb}}}{\tau_{m_{\text{Mfb}}}}$ | $\frac{dj_{\text{Mfb}}}{dt}=\frac{\bar{j}_{\text{Mfb}}-j_{\text{Mfb}}}{\tau_{j_{\text{Mfb}}}}$ |
| $\tau_{m_{\text{Mfb}}}=\frac{1}{\alpha_{m_{\text{Mfb}}}+\beta_{m_{\text{Mfb}}}}$ | $\tau_{j_{\text{Mfb}}}=\frac{1}{\alpha_{j_{\text{Mfb}}}+\beta_{j_{\text{Mfb}}}}$ |
| $\alpha_{m_{\text{Mfb}}}=\left\{ \begin{aligned} 0.0077\frac{V_{\text{M}\text{fb}}+68.19}{1.0-e^{-\text{0.18}\left( V_{\text{M}\text{fb}}+68.19 \right)}} \\ 0.0433, \text{if} V_{\text{M}\text{fb}}=-68.19 \end{aligned} \right.$ | |
| $\beta_{m_{\text{Mfb}}}=0.004e^{-V_{\text{M}\text{fb}}/\text{11.98}}$ | |
| $\alpha_{j_{\text{Mfb}}}=\left\{ \begin{aligned} \left( -14.5e^{\text{0.17}V_{\text{M}\text{fb}}}+1.8e^{\text{1.56}V_{\text{M}\text{fb}}} \right)\cdot\frac{V_{\text{M}\text{fb}}+35.73}{1.0+e^{\text{3.31}\left( V_{\text{M}\text{fb}}+3.86 \right)}} \\ 0, \text{if} V_{\text{M}\text{fb}}\geq-40.0 \end{aligned} \right.$ | |
| $\beta_{j_{\text{Mfb}}}=\left\{ \begin{aligned} 5.05\times{10}^{\text{-5}}\frac{e^{-\text{0.0995}V_{\text{M}\text{fb}}}}{1.0+e^{-\text{0.01}\left( V_{\text{M}\text{fb}}-84.02 \right)}} \\ 0.11\frac{e^{-4.13\times{10}^{\text{-}\text{4}}V_{\text{M}\text{fb}}}}{1.0+e^{-\text{0.09}\left( V_{\text{M}\text{fb}}+42.44 \right)}}, \text{if} V_{\text{M}\text{fb}}\geq-40.0 \end{aligned} \right.$ | |
| $E_{\text{Na,Mfb}}=\frac{\text{RT}}{\text{F}}\text{log}\frac{{[\text{Na}^{+}]}_{\text{c,Mfb}}}{{[\text{Na}^{+}]}_{\text{i,Mfb}}}$ |  |

**Table S21. Mechano-gated channel mediated current: *I*_MGC_Mfb_**

| $I_{\text{MGC\_Mfb}}=\bar{g}_{\text{MGC,Mfb}}\cdot\left( V_{\text{M}\text{fb}}-E_{\text{MGC,Mfb}} \right)$ |
| --- |

**Table S22. Intracellular ion concentrations: [Na^+^]_i,Mfb_, [K^+^]_i,Mfb_, and [Ca^2+^]_i,Mfb_**

| Without *I*_Na_Mfb_: $\frac{{d\left[ \text{Na}^{+} \right]}_{\text{i,Mfb}}}{dt}=-\frac{I_{\text{B,Na\_Mfb}}+{3I}_{\text{NaK\_Mfb}}}{\text{Vol}_{\text{i}\text{,Mfb}}\text{F}}$ |
| --- |
| With *I*_Na_Mfb_: $\frac{{d\left[ \text{Na}^{+} \right]}_{\text{i,Mfb}}}{dt}=-\frac{I_{\text{Na}\text{\_Mfb}}+I_{\text{B,Na\_Mfb}}+{3I}_{\text{NaK\_Mfb}}}{\text{Vol}_{\text{i}\text{,Mfb}}\text{F}}$ |
| $\frac{{d\left[ \text{K}^{+} \right]}_{\text{i,Mfb}}}{dt}=-\frac{I_{\text{K1\_Mfb}}+I_{\text{Kv\_Mfb}}}{\text{Vol}_{\text{i}\text{,Mfb}}\text{F}}$ |

**Mfb-M electrical coupling**

**Table S23. Transmembrane potential of myocyte and Mfb**

| $\frac{{dV}_{\text{M}}}{dt}=-\frac{1}{\text{C}_{\text{m,M}}}\left( I_{\text{M}}\left( V_{\text{M}},t \right)+\sum_{\text{i=1}}^{\text{n}} G_{\text{gap}}\left( V_{\text{M}}-V_{\text{M}\text{fb,i}} \right) \right)$ |
| --- |
| $\frac{{dV}_{\text{M}\text{fb,i}}}{dt}=-\frac{1}{\text{C}_{\text{m,Mfb}}}\left( I_{\text{Mfb,i}}\left( V_{\text{M}\text{fb,i}},t \right)+G_{\text{gap}}\left( V_{\text{M}\text{fb,i}}-V_{\text{M}} \right) \right)$ |
| Without *I*_SAC_: $I_{\text{M}}\left( V_{\text{M}},t \right)=$  $I_{\text{Na}}+I_{\text{CaL}}+I_{\text{t}}+I_{\text{Kur}}+I_{\text{K1}}+I_{\text{K,r}}+I_{\text{K,s}}+I_{\text{B,Na}}+I_{\text{B,Ca}}+I_{\text{NaK}}+I_{\text{CaP}}+I_{\text{NaCa}}-I_{\text{Stim}}$ |
| With *I*_SAC_: $I_{\text{M}}\left( V_{\text{M}},t \right)=$  $I_{\text{Na}}+I_{\text{CaL}}+I_{\text{t}}+I_{\text{Kur}}+I_{\text{K1}}+I_{\text{K,r}}+I_{\text{K,s}}+I_{\text{B,Na}}+I_{\text{B,Ca}}+I_{\text{NaK}}+I_{\text{CaP}}+I_{\text{NaCa}}+I_{\text{SAC}}-I_{\text{Stim}}$ |
| Without *I*_Na_Mfb_ and *I*_MGC_Mfb_: $I_{\text{Mfb,i}}\left( V_{\text{M}\text{fb,i}},t \right)=I_{\text{Kv}\text{\_Mfb}}+I_{\text{K}\text{1\_Mfb}}+I_{\text{NaK\_Mfb}}+I_{\text{B,Na\_Mfb}}$ |
| With *I*_Na_Mfb_ and *I*_MGC_Mfb_: $I_{\text{Mfb,i}}\left( V_{\text{M}\text{fb,}\text{I}},t \right)=$  $I_{\text{Kv}\text{\_Mfb}}+I_{\text{K}\text{1\_Mfb}}+I_{\text{NaK\_Mfb}}+I_{\text{B,Na\_Mfb}}+I_{\text{Na\_Mfb}}+I_{\text{MGC\_Mfb}}$ |

**Mechanical modeling of a single segment**

The mechanical modeling of a single segment is based on the classical three-element rheological scheme [[3](#_ENREF_3), [9](#_ENREF_9)].

**Table S24. The classical three-element rheological scheme**

| $F_{\mathrm{CE}}=f_{\mathrm{CE}}\cdot f_{v}\left( v \right){\cdot F}_{\mathrm{norm}}$ |
| --- |
| $f_{v}\left( v \right)=\frac{1-\left( \frac{v}{v_{\max}} \right)}{1+c_{v}\left( \frac{v}{v_{\max}} \right)}$ |
| $F_{\mathrm{SE}}=f_{\mathrm{SE}}\left[ e^{k_{\mathrm{SE}}\left( l_{\mathrm{SE}}-l_{\mathrm{SE}_{0}} \right)}-1 \right]$ |
| $F_{\mathrm{PE}}=f_{\mathrm{PE}}\left[ e^{k_{\mathrm{PE}}\left( l_{\mathrm{PE}}-l_{\mathrm{PE}_{0}} \right)}-1 \right]$ |
| $F_{\mathrm{CE}}=F_{\mathrm{SE}}$ |
| $F_{\mathrm{segment}}=F_{\mathrm{SE}}+F_{\mathrm{PE}}$ |
| $l_{\mathrm{PE}}=l_{\mathrm{CE}}+l_{\mathrm{SE}}$ |

**Table S25. Parameter values**

| [Na^+^]_b_ = 130.0 mM | $k_{\text{CaP}}$ = 0.0002 mM |
| --- | --- |
| [K^+^]_b_ = 5.4 mM | $k_{\text{Na}\text{Ca}}$ = 0.0374842 pA/(mM)^4^ |
| [Ca^2+^]_b_ = 1.8 mM | *γ* = 0.45 |
| [Mg^2+^]_i_ = 2.5 mM | *d*_NaCa_ = 0.0003 (mM)^-4^ |
| *E*_Ca,app_ = 60.0 mV | $\bar{I}_{\text{up}}$ = 2800.0 pA |
| *k*_Ca_ = 0.025 mM | *k*_cyca_ = 0.0003 mM |
| R = 8314.0 mJ/molK | *K*_srca_ = 0.5 mM |
| T = 306.15 K | *K*_xcs_ = 0.4 |
| F = 96487.0 C/mol | *τ*_tr_ = 0.01 s |
| C_m,M_ = 0.05 nF | *α*_rel_ = 200000.0 pA (mM)^-1^ |
| Vol_i_ = 0.005884 nL | *k*_rel,i_ = 0.0003 mM |
| Vol_c_ = 0.136Vol_i_ | *k*_rel,d_ = 0.003 mM |
| Vol_d_ = 0.02Vol_i_ | *r*_recov_ = 0.815 s^-1^ |
| Vol_rel_ = 0.0000441 nL | *K*_SAC_ = 100 |
| Vol_up_ = 0.0003969 nL | *α*_SAC_ = 3 |
| *τ*_Na_ = 14.3 s | *λ* = 1.2 |
| *τ*_K_ = 10.0 s | C_m,Mfb_ = 6.3 pF |
| *τ*_Ca_ = 24.7 s | Vol_i,Mfb_ = 0.00137 nL |
| *τ*_dt_ = 0.01 s | [Na^+^]_c,Mfb_ = 130.011 mM |
| $\bar{I}_{\text{NaK}}$ = 68.55 pA | [K^+^]_c,Mfb_ = 5.3581 mM |
| $k_{\text{NaK}\text{,K}}$ = 1.0 mM | *k*_mK_ = 1.0 mmol |
| $k_{\text{NaK}\text{,Na}}$ = 11.0 mM | *k*_mNa_ = 11.0 mmol |
| $\bar{I}_{\text{CaP}}$ = 4.0 pA | $\bar{I}_{\text{NaK}\text{,Mfb}}$ = 10.36 pA |
| [LTRPN]_tot_ = 70.0 μmol/L | *f*_CE_ = 100 mN/mm^2^ |
| [HTRPN]_tot_ = 140.0 μmol/L | *v*_max_ = 0.0055 μm/ms |
| $k_{\mathrm{ltrpn}}^{+}$ = 20.0 mol/L^-1^⋅s^-1^ | *c_v_* = 2 |
| $k_{\mathrm{ltrpn}}^{-}$ = 40.0 s^-1^ | *f*_SE_ = 2.8 mN/mm^2^ |
| $k_{\mathrm{htrpn}}^{+}$ = 1.0×10^8^ mol/L^-1^⋅s^-1^ | *k*_SE_ = 14.6 μm^-1^ |
| $k_{\mathrm{htrpn}}^{-}$ = 0.33 s^-1^ | *l*_SE0_ = 0 μm |
| SL = 1.78 μm | *f*_PE_ = 0.006 mN/mm^2^ |
| $\zeta$ = 0.1 N/mm^2^ | *k*_PE_ = 14.6 μm^-1^ |
| *f*_XB_ = 10 s^-1^ | *l*_PE0_ = 1.78 μm |
| $g_{\mathrm{XB}}^{*}$ = 30 s^-1^ |  |

**Table S26. Maximum conductance values**

| *P*_Na_ = 0.0018 nL/s | $\bar{g}_{\text{B,Ca}}$ = 0.078681 nS |
| --- | --- |
| $\bar{g}_{\text{CaL}}$ = 6.75 nS | $G_{\text{SAC}}$ = 0.015 μm/s |
| $\bar{g}_{\text{t}}$ = 8.25 nS | $\bar{g}_{\text{Kv}\text{,Mfb}}$ = 1.575 nS |
| $\bar{g}_{\text{Kur}}$ = 2.25 nS | $\bar{g}_{\text{K1}\text{,Mfb}}$ = 3.038 nS |
| $\bar{g}_{\text{K,s}}$ = 1.0 nS | $\bar{g}_{\text{B,Na,Mfb}}$ = 0.05985 nS |
| $\bar{g}_{\text{K,r}}$ = 0.5 nS | $\bar{g}_{\text{Na}\text{,Mfb}}$ = 0.756 nS |
| $\bar{g}_{\text{K1}}$ = 3.1 nS | $\bar{g}_{\text{MGC,Mfb}}$ = 0.043 nS |
| $\bar{g}_{\text{B,Na}}$ = 0.060599 nS |  |

**Table S27. Initial conditions**

| *V*_M_ = -74.2525 mV | *s*_Kur_ = 0.9673 |
| --- | --- |
| [Na^+^]_c_ = 130.0221 mM | *n* = 4.374×10^-3^ |
| [K^+^]_c_ = 5.5602 mM | *p*_a_ = 5.3×10^-5^ |
| [Ca^2+^]_c_ = 1.8158 mM | *F*_1_ = 0.4701 |
| [Na^+^]_i_ = 8.5168 mM | *F*_2_ = 0.0028 |
| [K^+^]_i_ = 129.486 mM | *O* = 1.382 |
| [Ca^2+^]_i_ = 6.5×10^-5^ mM | *O*_C_ = 0.0268 |
| [Ca^2+^]_d_ = 7.1×10^-5^ mM | *O*_Calse_ = 0.4315 |
| [Ca^2+^]_up_ = 0.6492 mM | *O*_TC_ = 0.0129 |
| [Ca^2+^]_rel_ = 0.6326 mM | *O*_TMgC_ = 0.1904 |
| *m* = 3.289×10^-3^ | *O*_TMgMg_ = 0.7145 |
| *h*_1_ = 0.8772 | [K^+^]_i,Mfb_ = 129.4349 mM |
| *h*_2_ = 0.8739 | [Na^+^]_i,Mfb_ = 8.5547 mM |
| *d*_L_ = 1.4×10^-5^ | *V*_Mfb_ = -47.75 mV |
| *f*_L1_ = 0.9986 | *r*_Kv_ = 0.0743 |
| *f*_L2_ = 0.9986 | *s*_Kv_ = 0.9717 |
| *r* = 1.089×10^-3^ | *m*_Mfb_ = 3.0×10^-3^ |
| *s* = 0.9486 | *j*_Mfb_ = 0.9989 |
| *r*_Kur_ = 3.67×10^-4^ | [LTRPNCa] = 0.4068232 μmol/L |
| [HTRPNCa] = 0.132944 mM | *P*1 = 1.47026×10^-6^ |
| *N*0 = 0.99999027 | *P*2 = 1.83527×10^-6^ |
| *N*1 = 3.88506×10^-6^ | *P*3 = 1.06786×10^-6^ |
| *P*0 = 1.46758×10^-6^ | *l*_CE_ = 1.78 μm |
| *l*_PE_ = 1.78 μm | *F*_PE_ = 10.0 mN/mm^2^ (in isotonic contraction) |

**Glossary**

| **Myocyte** | | *K*_xcs_ | Ratio of forward to back reactions for *I*_up_ | |
| --- | --- | --- | --- | --- |
| *I*_Na_ | Na^+^ current | *τ*_tr_ | Time constant of diffusion of Ca^2+^ from sarcoplasmic reticulum uptake to release compartment | |
| *I*_CaL_ | L-type Ca^2+^ current | *α*_rel_ | Scaling factor for *I*_rel_ | |
| *I*_t_ | Transient outward K^+^ current | *k*_rel,i_ | Half-activation [Ca^2+^]_i_ for *I*_rel_ | |
| *I*_Kur_ | Sustained outward K^+^ current | *k*_rel,d_ | Half-activation [Ca^2+^]_d_ for *I*_rel_ | |
| *I*_K,s_ | Slow delayed rectifier K^+^ current | *k*_recov_ | Recovery rate constant for the sarcoplasmic reticulum release channel | |
| *I*_K,r_ | Rapid delayed rectifier K^+^ current | *I*_SAC_ | Stretch-activated current | |
| *I*_K1_ | Inwardly rectifying K^+^ current | *I*_SAC,Na_ | Na^+^ contributes to *I*_sac_ | |
| *I*_B,Na_ | Background Na^+^ current | *I*_SAC,K_ | K^+^ contributes to *I*_sac_ | |
| *I*_B,Ca_ | Background Ca^2+^ current | *I*_SAC,Ca_ | Ca^2+^ contributes to *I*_sac_ | |
| *I*_NaK_ | Na^+^-K^+^ pump current | $\bar{P}_{\text{Na}}$ | Relative permeability to Na^+^ | |
| *I*_CaP_ | Sarcolemmal Ca^2+^ pump current | $\bar{P}_{\text{K}}$ | Relative permeability to K^+^ | |
| *I*_NaCa_ | Na^+^-Ca^2+^ exchange current | $\bar{P}_{\text{C}\text{a}}$ | Relative permeability to Ca^2+^ | |
| *I*_di_ | Ca^2+^ diffusion current from the diffusion-restricted subsarcolemmal space to the cytosol | *z*_Na_ | Na^+^ valence | |
| *I*_up_ | Sarcoplasmic reticulum Ca^2+^ uptake current | *z*_K_ | K^+^ valence | |
| *I*_tr_ | Sarcoplasmic reticulum Ca^2+^ translocation current (from uptake to release compartment) | *z*_Ca_ | Ca^2+^ valence | |
| *I*_rel_ | Sarcoplasmic reticulum Ca^2+^ release current | *g*_SAC_ | Conductance for *I*_sac_ | |
| [Na^+^]_b_ | Na^+^ concentration in bulk (bathing) medium | *G*_SAC_ | Maximum conductance for *I*_sac_ | |
| [K^+^]_b_ | K^+^ concentration in bulk (bathing) medium | *K*_SAC_ | Parameter to define the amount of current when the cell is not stretched | |
| [Ca^2+^]_b_ | Ca^2+^ concentration in bulk (bathing) medium | *α*_SAC_ | Parameter to describe the sensitivity to stretch | |
| [Na^+^]_c_ | Na^+^ concentration in the extracellular cleft space | *λ* | Stretch ratio | |
| [K^+^]_c_ | K^+^ concentration in the extracellular cleft space | [LTRPN]_tot_ | Total troponin low-affinity site concentration | |
| [Ca^2+^]_c_ | Ca^2+^ concentration in the extracellular cleft space | [HTRPN]_tot_ | Total troponin high-affinity site concentration | |
| [Na^+^]_i_ | Na^+^ concentration in the intracellular medium | $k_{\mathrm{ltrpn}}^{+}$ | Ca^2+^ on-rate for troponin low affinity sites | |
| [K^+^]_i_ | K^+^ concentration in the intracellular medium | $k_{\mathrm{ltrpn}}^{-}$ | Ca^2+^ off-rate for troponin low affinity sites | |
| [Ca^2+^]_i_ | Ca^2+^ concentration in the intracellular medium | $k_{\mathrm{htrpn}}^{+}$ | Ca^2+^ on-rate for troponin high affinity sites | |
| [Mg^2+^]_i_ | Mg^2+^ concentration in the intracellular medium | $k_{\mathrm{htrpn}}^{-}$ | Ca^2+^ off-rate for troponin high affinity sites | |
| [Ca^2+^]_d_ | Ca^2+^ concentration in the restricted subsarcolemmal space | [LTRPNCa] | Concentration of Ca^2+^ bound to low-affinity troponin sites | |
| [Ca^2+^]_up_ | Ca^2+^ concentration in the sarcoplasmic reticulum uptake compartment | [HTRPNCa] | Concentration of Ca^2+^ bound to high-affinity troponin sites | |
| [Ca^2+^]_rel_ | Ca^2+^ concentration in the sarcoplasmic reticulum release compartment | $\zeta$ | Conversion factor for normalization to physiological force | |
| *E*_Na_ | Equilibrium (Nernst) potential for Na^+^ | *f*_XB_ | Basic transition rate from weak to strong cross bridge | |
| *E*_K_ | Equilibrium (Nernst) potential for K^+^ | $g_{XB}^{*}$ | Minimum transition rate from strong to weak cross bridge | |
| *E*_Ca_ | Equilibrium (Nernst) potential for Ca^2+^ | SL | Sarcomere length | |
| *E*_Ca,app_ | Apparent reversal potential for *I*_CaL_ | *N*0 | Nonpermissive tropomyosin with 0 cross bridges | |
| *P*_Na_ | Permeability for *I*_Na_ | *N*1 | Nonpermissive tropomyosin with 1 cross bridge | |
| $\bar{g}_{\text{CaL}}$ | Maximum conductance for *I*_CaL_ | *P*0 | Permissive tropomyosin with 0 cross bridges | |
| $\bar{g}_{\text{t}}$ | Maximum conductance for *I*_t_ | *P*1 | Permissive tropomyosin with 1 cross bridge | |
| $\bar{g}_{\text{Kur}}$ | Maximum conductance for *I*_Kur_ | *P*2 | Permissive tropomyosin with 2 cross bridges | |
| $\bar{g}_{\text{K,s}}$ | Maximum conductance for *I*_K,s_ | *P*3 | Permissive tropomyosin with 3 cross bridges | |
| $\bar{g}_{\text{K,r}}$ | Maximum conductance for *I*_K,r_ | *F*_norm_ | Normalized force | |
|  |  |  |  | |
| $\bar{g}_{\text{K1}}$ | Maximum conductance for *I*_K1_ |  | | |
| $\bar{g}_{\text{B,Na}}$ | Maximum conductance for *I*_B,Na_ | **Myofibroblast** | | |
| $\bar{g}_{\text{B,Ca}}$ | Maximum conductance for *I*_B,Ca_ | *I*_Kv_Mfb_ | Time- and voltage-dependent K^+^ current | |
| *m* | Activation gating variable for *I*_Na_ | *I*_K1_Mfb_ | Inward-rectifying K^+^ current | |
| *h*_1_, *h*_2_ | Fast and slow inactivation gating variables for *I*_Na_ | *I*_NaK_Mfb_ | Na^+^-K^+^ pump current | |
| *d*_L_ | Activation gating variable for *I*_CaL_ | *I*_B,Na_Mfb_ | Background Na^+^ current | |
| *f*_L1_, *f*_L2_ | Fast and slow inactivation gating variables for *I*_CaL_ | *I*_Na_Mfb_ | Na^+^ current | |
| *f*_Ca_ | [Ca^2+^]_d_-dependent ratio of fast (*f*_L1_) to slow (*f*_L2_) inactivation of *I*_CaL_ | *I*_MGC_Mfb_ | Mechano-gated current | |
| *k*_Ca_ | Half-maximum Ca^2+^ binding concentration for *f*_Ca_ | [Na^+^]_c,Mfb_ | Na^+^ concentration in the extracellular cleft space | |
| *r* | Activation gating variable for *I*_t_ | [K^+^]_c,Mfb_ | K^+^ concentration in the extracellular cleft space | |
| *s* | Inactivation gating variable for *I*_t_ | [Na^+^]_i,Mfb_ | Na^+^ concentration in the intracellular medium | |
| *s*_1_, *s*_2_ | Rapidly and slowly recovering inactivation gating variables for *I*_t_ | [K^+^]_i,Mfb_ | K^+^ concentration in the intracellular medium | |
| *r*_Kur_ | Activation gating variable for *I*_Kur_ | *E*_Na,Mfb_ | Equilibrium (Nernst) potential for Na^+^ | |
| *s*_Kur_ | Inactivation gating variable for *I*_Kur_ | *E*_K,Mfb_ | Equilibrium (Nernst) potential for K^+^ | |
| *n* | Activation gating variable for *I*_K,s_ | *E*_MGC,Mfb_ | Equilibrium (Nernst) potential for ion through mechano-gate channels | |
| *p*_a_ | Activation gating variable for *I*_K,r_ | $\bar{g}_{\text{Kv,Mfb}}$ | Maximum conductance for *I*_Kv_Mfb_ | |
| *p*_i_ | Inactivation gating variable (instantaneous) for *I*_K,r_ | $\bar{g}_{\text{K1,Mfb}}$ | Maximum conductance for *I*_K1_Mfb_ | |
| $\bar{m}, \bar{h}_{\text{1}},\cdots$ | Steady-state value of *m*, *h*_1_, etc | $\bar{g}_{\text{B,Na,Mfb}}$ | Maximum conductance for *I*_b,Na_Mfb_ | |
| *F*_1_ | Relative amount of “inactive precursor” in the *I*_rel_ formulation | $\bar{g}_{\text{Na,Mfb}}$ | Maximum conductance for *I*_Na_Mfb_ | |
| *F*_2_ | Relative amount of “activator” in the *I*_rel_ formulation | $\bar{g}_{\text{MGC,Mfb}}$ | Maximum conductance for *I*_MGC_Mfb_ | |
| *τ*_m_ | Activation time constant for *I*_Na_ | *r*_Kv_ | Activation gating variable for *I*_Kv_Mfb_ | |
| $\tau_{h_{\text{1}}}, \tau_{h_{\text{2}}}$ | Fast and slow inactivation time constants for *I*_Na_ | *S*_Kv_ | Inactivation gating variable for *I*_Kv_Mfb_ | |
| $\tau_{d_{\text{L}}}$ | Activation time constant for *I*_CaL_ | $\tau_{r_{\text{Kv}}}$ | Activation time constant for *I*_Kv_Mfb_ | |
| $\tau_{f_{\text{L1}}}, \tau_{f_{\text{L2}}}$ | Fast and slow inactivation time constants for *I*_CaL_ | $\tau_{s_{\text{Kv}}}$ | Inactivation time constant for *I*_Kv_Mfb_ | |
| *τ*_r_ | Activation time constant for *I*_t_ | *α*_K1_, *β*_K1_ | Fractional open probability of the *I*_K1_Mfb_ channel | |
| *τ*_s_ | Inactivation time constant for *I*_t_ | $\bar{I}_{\text{NaK}\text{,max}}$ | Maximum Na^+^-K^+^ pump current | |
| $\tau_{r_{\text{Kur}}}$ | Activation time constant for *I*_Kur_ | *k*_mK_ | Half-maximum K^+^ binding concentration for *I*_NaK_ | |
| $\tau_{s_{\text{Kur}}}$ | Inactivation time constant for *I*_Kur_ | *k*_mNa_ | Half-maximum Na^+^ binding concentration for *I*_NaK_ | |
| *τ*_n_ | Activation time constant for *I*_K,s_ | *m*_Mfb_ | Activation gating variable for *I*_Na_Mfb_ | |
| $\tau_{p_{\text{a}}}$ | Activation time constant for *I*_K,r_ | *j*_Mfb_ | Inactivation gating variable for *I*_Na_Mfb_ | |
| *O* | Buffer occupancy | $\tau_{m_{\text{Mfb}}}$ | Activation time constant for *I*_Na_Mfb_ | |
| *O*_C_ | Fractional occupancy of the calmodulin buffer by Ca^2+^ | $\tau_{j_{\text{Mfb}}}$ | Inactivation time constant for *I*_Na_Mfb_ | |
| *O*_TC_ | Fractional occupancy of the troponin-Ca^2+^ buffer by Ca^2+^ | ${}_{m_{\text{Mfb}}},{}_{j_{\text{Mfb}}},$  ${}_{m_{\text{Mfb}}},{}_{j_{\text{Mfb}}}$ | Extrapolated rate coefficients | |
| *O*_TMgC_ | Fractional occupancy of the troponin-Mg^2+^ buffer by Ca^2+^ | *E*_Na,Mfb_ | Equilibrium (Nernst) potential for Na^+^ | |
| *O*_TMgMg_ | Fractional occupancy of the troponin-Mg^2+^ buffer by Mg^2+^ | *E*_K,Mfb_ | Equilibrium (Nernst) potential for K^+^ | |
| *O*_Calse_ | Fractional occupancy of the calsequestrin buffer (in the sarcoplasmic reticulum release compartment) by Ca^2+^ | *E*_MGC,Mfb_ | Equilibrium (Nernst) potential for ion through mechano-gate channels | |
| R | Universal gas constant | $\bar{r}_{Kv}, \bar{s}_{Kv},\cdots$ | Steady-state value of *r*_Kv_, *s*_Kv_, etc | |
| T | Absolute temperature | C_m,Mfb_ | Membrane capacitance | |
| F | Faraday’s constant | *V*_Mfb_ | Membrane voltage | |
| C_m,M_ | Membrane capacitance |  | |  |
| *V*_M_ | Membrane voltage | **Mfb-M coupling** | | |
| Vol_c_ | Volume of the extracellular cleft space | *G*_gap_ | Gap junctional conductance between Mfb and myocyte | |
| Vol_i_ | Total cytosolic volume | n | Number of Mfbs per myocyte | |
| Vol_d_ | Volume of the diffusion-restricted subsarcolemmal space | *I*_M_ | Net membrane current of the myocyte | |
| Vol_up_ | Volume of the sarcoplasmic reticulum uptake compartment | *I*_Mfb_ | Net membrane current of the Mfb | |
| Vol_rel_ | Volume of the sarcoplasmic reticulum release compartment | **Segment mechanics** | | |
| *τ*_Na_,  *τ*_K_,*τ*_Ca_, | Time constant of diffusion of Na^+^, K^+^, and Ca^2+^ from the bulk medium to the extracellular cleft space | *f*_CE_ | Scaling factor for contractile element | |
| *τ*_di_ | Time constant of diffusion from the restricted subsarcolemmal space to the cytosol | *v*_max_ | Maximum sarcomere shortening velocity | |
| Vol_i_ | Total cytosolic volume | *c_v_* | Constant describing the shape of the hyperbole | |
| $\bar{I}_{\text{NaK}}$ | Maximum Na^+^-K^+^ pump current | *f*_SE_ | Scaling factor for series elastic element | |
| *k*_NaK,K_ | Half-maximum K^+^ binding concentration for *I*_NaK_ | *k*_SE_ | Material constant for series elastic element | |
| *k*_NaK,Na_ | Half-maximum Na^+^ binding concentration for *I*_NaK_ | *l*_SE_ | Actual length of series elastic element | |
| $\bar{I}_{\text{CaP}}$ | Half-maximum Ca2+ binding concentration for *I*_CaP_ | *l*_SE0_ | Reference length of series elastic element | |
| *k*_NaCa_ | Scaling factor for *I*_NaCa_ | *f*_PE_ | Scaling factor for parallel elastic element | |
| *γ* | Position of energy barrier controlling voltage dependence of *I*_NaCa_ | *k*_PE_ | Material constant for parallel elastic element | |
| *d*_NaCa_ | Denominator constant for *I*_NaCa_ | *l*_PE_ | Actual length of parallel elastic element | |
| $\bar{I}_{\text{up}}$ | Maximum sarcoplasmic reticulum uptake current | *l*_PE0_ | Reference length of parallel elastic element | |
| *k*_cyca_ | Half-maximum binding concentration for [Ca^2+^]_i_ to *I*_up_ | *f*_CE_ | Scaling factor for contractile element | |
| *k*_srca_ | Half-maximum binding concentration for [Ca^2+^]_up_ to *I*_up_ |  |  | |

**References**

1. Maleckar MM, Greenstein JL, Giles WR, Trayanova NA: **K+ current changes account for the rate dependence of the action potential in the human atrial myocyte.** *Am J Physiol Heart Circ Physiol* 2009, **297**(4):H1398-1410.

2. Nygren A, Fiset C, Firek L, Clark JW, Lindblad DS, Clark RB, Giles WR: **Mathematical model of an adult human atrial cell: the role of K+ currents in repolarization.** *Circulation research* 1998, **82**(1):63-81.

3. Kuijpers NH, ten Eikelder HM, Bovendeerd PH, Verheule S, Arts T, Hilbers PA: **Mechanoelectric feedback leads to conduction slowing and block in acutely dilated atria: a modeling study of cardiac electromechanics.** *Am J Physiol Heart Circ Physiol* 2007, **292**(6):H2832-2853.

4. Kim D: **Novel cation-selective mechanosensitive ion channel in the atrial cell membrane.** *Circ Res* 1993, **72**(1):225-231.

5. Rice JJ, Jafri MS, Winslow RL: **Modeling short-term interval-force relations in cardiac muscle.** *Am J Physiol Heart Circ Physiol* 2000, **278**(3):H913-931.

6. MacCannell KA, Bazzazi H, Chilton L, Shibukawa Y, Clark RB, Giles WR: **A mathematical model of electrotonic interactions between ventricular myocytes and fibroblasts.** *Biophysical Journal* 2007, **92**(11):4121-4132.

7. Chatelier A, Mercier A, Tremblier B, Theriault O, Moubarak M, Benamer N, Corbi P, Bois P, Chahine M, Faivre JF: **A distinct de novo expression of Nav1.5 sodium channels in human atrial fibroblasts differentiated into myofibroblasts.** *J Physiol* 2012, **590**(17):4307-4319.

8. Kamkin A, Kirischuk S, Kiseleva I: **Single mechano-gated channels activated by mechanical deformation of acutely isolated cardiac fibroblasts from rats.** *Acta physiologica* 2010, **199**(3):277-292.

9. Solovyova O, Katsnelson L, Guriev S, Nikitina L, Protsenko Y, Routkevitch S, Markhasin V: **Mechanical inhomogeneity of myocardium studied in parallel and serial cardiac muscle duplexes: experiments and models.** *Chaos Soliton Fract* 2002, **13**(8):1685-1711.
